# Biomimetic Gradient Hydrogels Regulate Osteochondral Regeneration Microenvironment Remodeling via Spatiotemporal Programming Engineering

**DOI:** 10.1002/advs.76469

**Published:** 2026-07-08

**Authors:** Xiaolian Niu, Shengzhao Xiao, Di Huang, Xuesong Wang, Yanwei Cao, Xiaodan Sun, Nicholas Dunne, Xiaoming Li

**Affiliations:** ^1^ Key Laboratory For Biomechanics and Mechanobiology of Ministry of Education, Key Laboratory of Innovation and Transformation of Advanced Medical Devices of Ministry of Industry and Information Technology National Medical Innovation Platform for Industry‐Education Integration in Advanced Medical Devices (Interdiscipline of Medicine and Engineering) Beijing Advanced Innovation Center for Biomedical Engineering School of Biological Science and Medical Engineering, Beihang University Beijing China; ^2^ Department of Biomedical Engineering Research Center for Nano‐biomaterials & Regenerative Medicine College of Artificial Intelligence Taiyuan University of Technology Taiyuan China; ^3^ Department of Orthodontics Shanghai Ninth People's Hospital Shanghai Jiao Tong University School of Medicine College of Stomatology Shanghai Jiao Tong University National Center for Stomatology National Clinical Research Center for Oral Diseases Shanghai Key Laboratory of Stomatology Shanghai Research Institute of Stomatology Shanghai China; ^4^ Sports Medicine Service Beijing Jishuitan Hospital Capital Medical University Beijing China; ^5^ Key Laboratory of Advanced Materials of Ministry of Education Tsinghua University Beijing China; ^6^ Centre For Medical Engineering Research School of Mechanical and Manufacturing Engineering Dublin City University Dublin Ireland

**Keywords:** angiogenesis‐osteogenesis coupling, continuous gradient scaffold, immunomodulation, osteochondral regeneration, spatiotemporal programming

## Abstract

Osteochondral repair remains a critical challenge owing to conventional scaffolds’ inadequate dynamic mechanical adaptability, poor interfacial integration, inefficient cell recruitment, and lack of spatiotemporal regulation of bioactive cues. Inspired by native tissue hierarchical gradients and endogenous healing mechanisms, we developed an electric field‐driven continuous gradient hydrogel (GHZF4) via spatiotemporal programming. Integrating nanofiber‐reinforced self‐adaptive matrix, electric field‐induced nanofiber alignment, and ZIF‐8 nanocarrier‐mediated bioactive release, GHZF4 constructs compositional/structural/mechanical gradients. Its bone‐mimetic zone achieves burst release of PDGF‐BB and sustained release of BMP‐2 for vascularized osteogenesis, while the cartilage‐mimetic zone sustains TGF‐β3 release for chondrogenesis. In vitro, GHZF4 enhances macrophage M2 polarization, autologous stem cell recruitment, angiogenesis, and osteochondral differentiation. In rat/rabbit defect models, it enables seamless integration and functional repair, validated by micro‐CT, nanoindentation and histology. Transcriptomic analysis reveals the potential upregulation of signaling pathways associated with immunomodulation, angiogenesis, and osteochondral differentiation, which shows strong consistency with our in vitro functional outcomes and in vivo regenerative phenotypes. This nanofunctionalized gradient scaffold spatiotemporally couples key repair processes, providing a promising proof‐of‐concept strategy for cell‐free functional osteochondral repair.

## Graphical abstract

Electric field‑driven continuous gradient hydrogel GHZF4 achieves spatiotemporal growth factor release, modulates immune microenvironment, angiogenesis and osteochondral differentiation. It realizes cell‑free functional osteochondral repair in rat/rabbit models, offering a promising biomimetic strategy for gradient tissue regeneration.

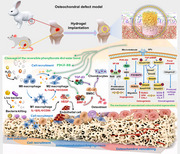

## 1 Introduction

Osteochondral defect repair remains clinically challenging due to inherent tissue heterogeneity, the absence of biomimetic transitional gradients, and insufficient dynamic mechanical adaptability to joint loading [1, 2, 3]. Conventional scaffolds fail to recapitulate native tissue's structural‐biochemical continuities, orchestrate stem cell recruitment, modulate inflammatory microenvironment or achieve spatiotemporally controlled angiogenesis, leading to poor interfacial integration and failed functional restoration [4, 5, 6].

The endogenous osteochondral healing mechanism is spatiotemporally ordered, relying on coordinated immunomodulation, autologous stem cell recruitment, and angiogenesis [7, 8, 9]. It initiates with inflammatory priming to clear necrotic debris, followed by stem cell recruitment from subchondral bone marrow and site‐specific differentiation, and concludes with subchondral angiogenesis (while preventing vascular invasion into cartilage) [3, 10]. Inspired by this mechanism, we propose several scaffold design requirements: (1) matching native tissue's continuous gradient microenvironment; (2) spatiotemporally regulating angiogenesis promoting in subchondral bone while inhibiting in cartilage; (3) guiding stem cell recruitment and zone‐specific differentiation; (4) modulating inflammatory microenvironment; (5) synchronizing immunomodulation, stem cell recruitment, and angiogenesis with endogenous healing stages via spatiotemporal programming.

Gradient scaffolds with spatially specific cues mimic complex biological signaling microenvironment [11, 12], and continuous gradient designs avoid interfacial delamination compared to bilayer/multilayer alternatives [3]. Hydrogels structurally analogous to the extracellular matrix serve as ideal carriers, with self‐healing properties adapting to complex joint defects and enabling stem cell migration [11, 12, 13, 14, 15, 16, 17, 18, 19]. To overcome the inherent limitations of conventional hydrogels, including inadequate dynamic adaptability, limited mechanical strength, and a lack of immunomodulatory and stem cell recruitment capabilities, we engineered nanofiber‐reinforced self‐adaptive hydrogels using polyvinyl alcohol (PVA), phenylboronic acid‐modified sodium alginate (SA‐PBA), and β‐sheet‐rich silk nanofibers (BSNF) via reversible PBA‐diol ester bonds [20]. Negatively charged BSNF migrate toward the anode under electric field, and cross‐linking immobilizes aligned nanofibers, generating biomimetic compositional/structural/mechanical gradients, core to our spatiotemporal programming strategy.

Precise spatiotemporal regulation of bioactive signals is essential for coordinating repair processes. Previous studies have demonstrated that the zonal organization and zone‐specific cellular phenotypes of native osteochondral tissue are regulated by spatiotemporal secretion of growth factors. Transforming growth factor‐β3 (TGF‐β3) dominates the progression of chondrocyte lineage differentiation, whereas bone morphogenetic protein‐2 (BMP‐2) acts as an indispensable regulator driving osteogenic maturation. In addition, platelet‐derived growth factor‐BB (PDGF‐BB) not only accelerates vascular network formation but also facilitates the directional recruitment of endogenous bone marrow mesenchymal stem cells (BMSC) toward damaged osteochondral lesion [21, 22, 23, 24, 25]. Spatially sequential co‐delivery of PDGF‐BB and BMP‐2 facilitates the formation of vascularized bone matrix [26, 27, 28, 29, 30], while PDGF‐BB/TGF‐β3 combination synergistically boosts stem cell homing and cartilaginous matrix deposition [31]. Our prior work also showed that ZIF‐8‐modified hydrogels enable sustained drug release for temporal programming [30]. Building on these findings, we hypothesized that spatially targeted incorporation of PDGF‐BB, BMP‐2, and TGF‐β3 into gradient hydrogels via ZIF‐8‐mediated release would achieve spatiotemporal programming of repair processes.

**FIGURE 1:**
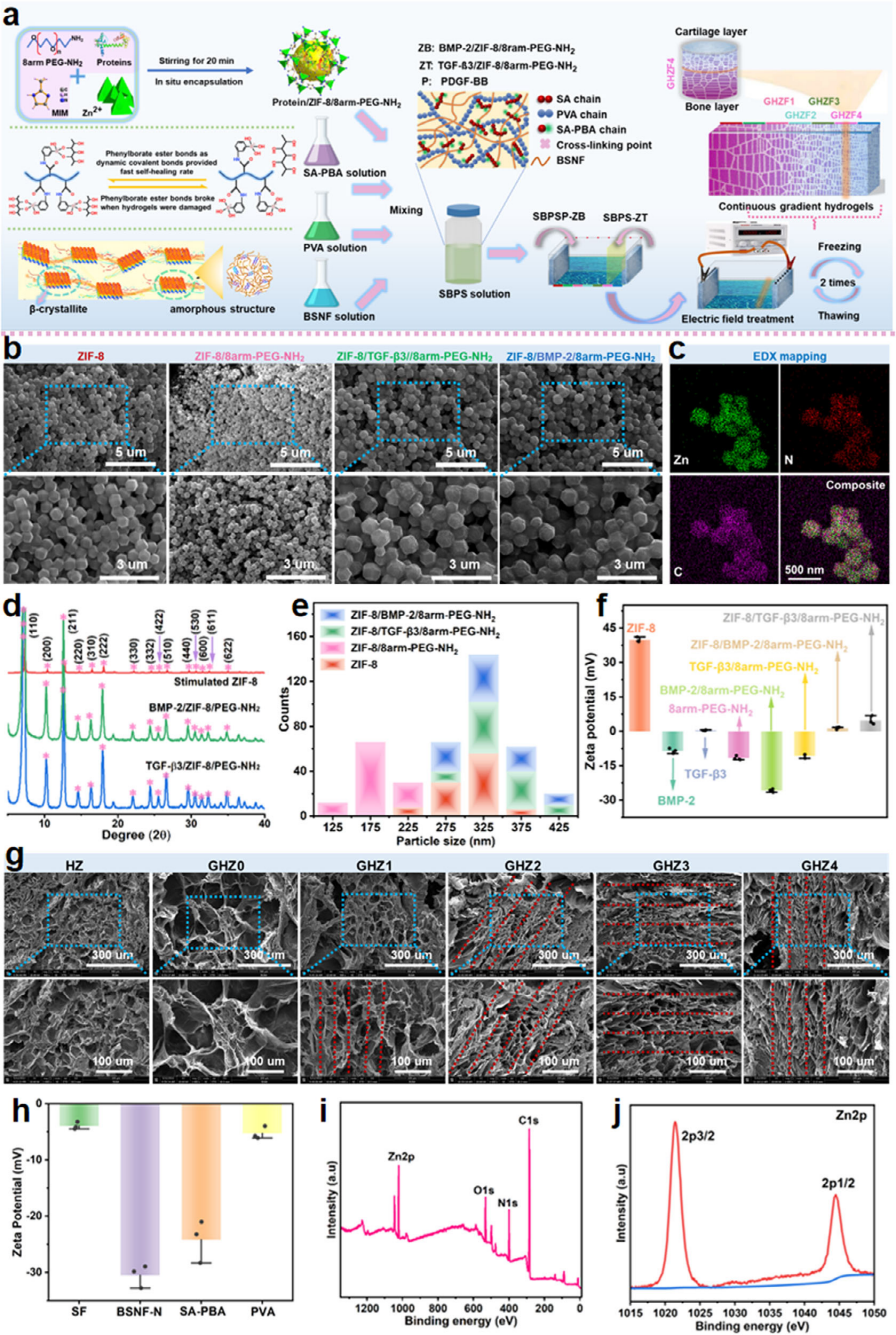
Fabrication and characterization of GFs‐loaded ZIF‐8 NPs and continuous gradient hydrogel. (a) The schematic fabrication process of continuous gradient hydrogel. (b) SEM images showing the morphology of NPs. (c) EDX mapping of BMP‐2/ZIF‐8/8arm‐PEG‐NH_2_ NPs. (d) XRD pattern of NPs. (e) The size distribution of NPs. f) Zeta potential of NPs. (g) The SEM images of the obtained continuous gradient composite hydrogel after freeze‐drying. (h) Zeta potential of the raw material used to synthesize the hydrogel. (i) XPS full spectra of ZIF‐8‐incorporated hydrogels. (j) High resolution of XPS Zn 2p spectra. *p < 0.05, **p < 0.01, ***p < 0.001.

Herein, we designed a continuous gradient hydrogel system using ZIF‐8 nanoparticles (NPs) for spatiotemporally controlled factor delivery (Figure 1a). Specifically, the bone‐like layer (SBPSP‐ZB) is fabricated by integrating ZIF‐8 NPs loaded with bioactive BMP‐2 into SBPS hydrogels pre‐embedded with PDGF‐BB, supporting osteoblast differentiation and subchondral angiogenesis. The cartilage‐like layer (SBPS‐ZT) integrates TGF‐β3‐functionalized ZIF‐8 NPs into SBPS hydrogels, guiding chondrogenesis and immunomodulation. These layers are seamlessly integrated via electric field and in situ cross‐linking. This scaffold combines nanofiber‐reinforced self‐adaptive mechanics, electric field‐driven gradient formation, and ZIF‐8‐mediated sustained factor release to orchestrate functional osteochondral repair.

## 2 Results

### 2.1 Preparation and Characterization of ZIF‐8/Protein/8arm‐PEG‐NH_2_ NPs

ZIF‐8/protein/8arm‐PEG‐NH_2_ NPs were synthesized via a one‐pot method using 2‐MIM and Zn(CH_3_COO)_2_·2H_2_O (Figure S1). SEM showed uniform rhombic dodecahedron morphology with consistent size (Figure 1b and Figure S2). Adding 8arm‐PEG‐NH_2_ competed for Zn^2+^ coordination, reducing mean particle size from 304.78 ± 33.26 to 181.87 ± 23.42 nm (Figure S3a). Encapsulation of BMP‐2/TGF‐β3 did not alter NP morphology (Figure 1e), and EDS elemental analysis verified that C, N, O, and Zn were homogeneously distributed over the entire surface of ZIF‐8/TGF‐β3/8arm‐PEG‐NH_2_ NPs (Figure 1c). The significant increase in particle size of ZIF‐8/BMP‐2/8arm‐PEG‐NH_2_ and ZIF‐8/TGF‐β3/8arm‐PEG‐NH_2_ is attributed to three factors: (1) BMP‐2/TGF‐β3 adsorb on ZIF‐8 via Zn^2^^+^ coordination and electrostatic interaction to form a thin protein shell; (2) loaded proteins slightly weaken the Zn^2^^+^‐competitive effect of 8arm‐PEG‐NH_2_ during synthesis; (3) weak intermolecular interactions induce mild reversible aggregation. XRD verified standard ZIF‐8 crystal structures, with surface modification enhancing crystallinity (Figure 1d). Zeta‐potentials shifted from 39.82 ± 1.12 mV (empty ZIF‐8) to 1.38 ± 0.54 mV (ZIF‐8/BMP‐2/8arm‐PEG‐NH_2_) and 4.71 ± 1.88 mV (ZIF‐8/TGF‐β3/8arm‐PEG‐NH_2_) due to the encapsulation of protein and 8arm‐PEG‐NH_2_, which partially neutralizes the surface positive charge of ZIF‐8 (Figure 1f). ELISA showed BMP‐2 and TGF‐β3 loading efficiencies of 5.48 ± 0.51% and 9.51 ± 0.44%, respectively (Figure S3b).

### 2.2 Fabrication of Electric Field‐Pattern Continuous Gradient Hydrogels

Self‐healing SBPS hydrogels were prepared with SA‐PBA:PVA = 6:4 and SBP (SA‐PBA+PVA):BSNF = 8:2^20^. For the bone layer precursor (SBPSP‐ZB), ZIF‐8 NPs conjugated with BMP‐2 were mixed into SBPS hydrogels preloaded with PDGF‐BB, while the chondral layer precursor (SBPS‐ZT) integrated TGF‐β3‐functionalized ZIF‐8 NPs into SBPS hydrogels. Precursor solutions were poured into opposite sides of an electrolytic cell. Upon power activation (50 V), BSNF migrated towards the anode and stagnated during the sol‐gel transition, forming a gradient distribution (Figure S4). Along the directional path stretching from cathode to anode, the hydrogel matrix displayed a continuous transition from high flexibility to high stiffness (Figure S5). For characterization, hydrogels were cut into seven sections along the electrical field direction and termed GHZF0, GHZF1, GHZF2, GHZF3, and GHZF4 from the cathode to the anode (Figure S4). GHZF0 was located adjacent to the cathode electrode and was affected by electrolysis bubbles, resulting in structural inhomogeneity and unreliable data. Thus, GHZF0 was excluded from all characterization and biological tests. Control groups included hydrogels without ZIF‐8/protein/8arm‐PEG‐NH_2_ NPs (GH4) and without electrical field treatment (HZF4). Detailed component ratios of hydrogels used in cellular and animal experiments are provided in Figure S6. The abbreviation “H” represents the SBPS hydrogel in this study.

### 2.3 Characterization of Electric Field‐Pattern Continuous Gradient Hydrogels

Given the extremely low dosage of growth factors (below 5 × 10^−5^ wt/vol%) incorporated within hydrogels, their influence on bulk physical performance is negligible. Thus, growth factors were excluded from hydrogel specimens to streamline sample fabrication.

Scanning electron microscopy (SEM) revealed an interconnected pore network, which enables sufficient cellular infiltration deep inside the scaffold matrix, with progressive increases in density, orientation, and network structure from GHZ0 to GHZ4 (Figure 1g). Zeta potentials of SA‐PBA, PVA, BSNF‐Z, and SF were −24.19 ± 3.74, −5.29 ± 1.34, −30.52 ± 2.01, and −3.95 ± 0.65 mV, respectively (Figure 1h). XPS confirmed C, O, N, and Zn elements, with Zn 2p peaks at 1044.48 and 1021.38 eV (Zn^2+^ 2p1/2 and Zn^2+^ 2p3/2), respectively (Figure 1i,j).

**FIGURE 2:**
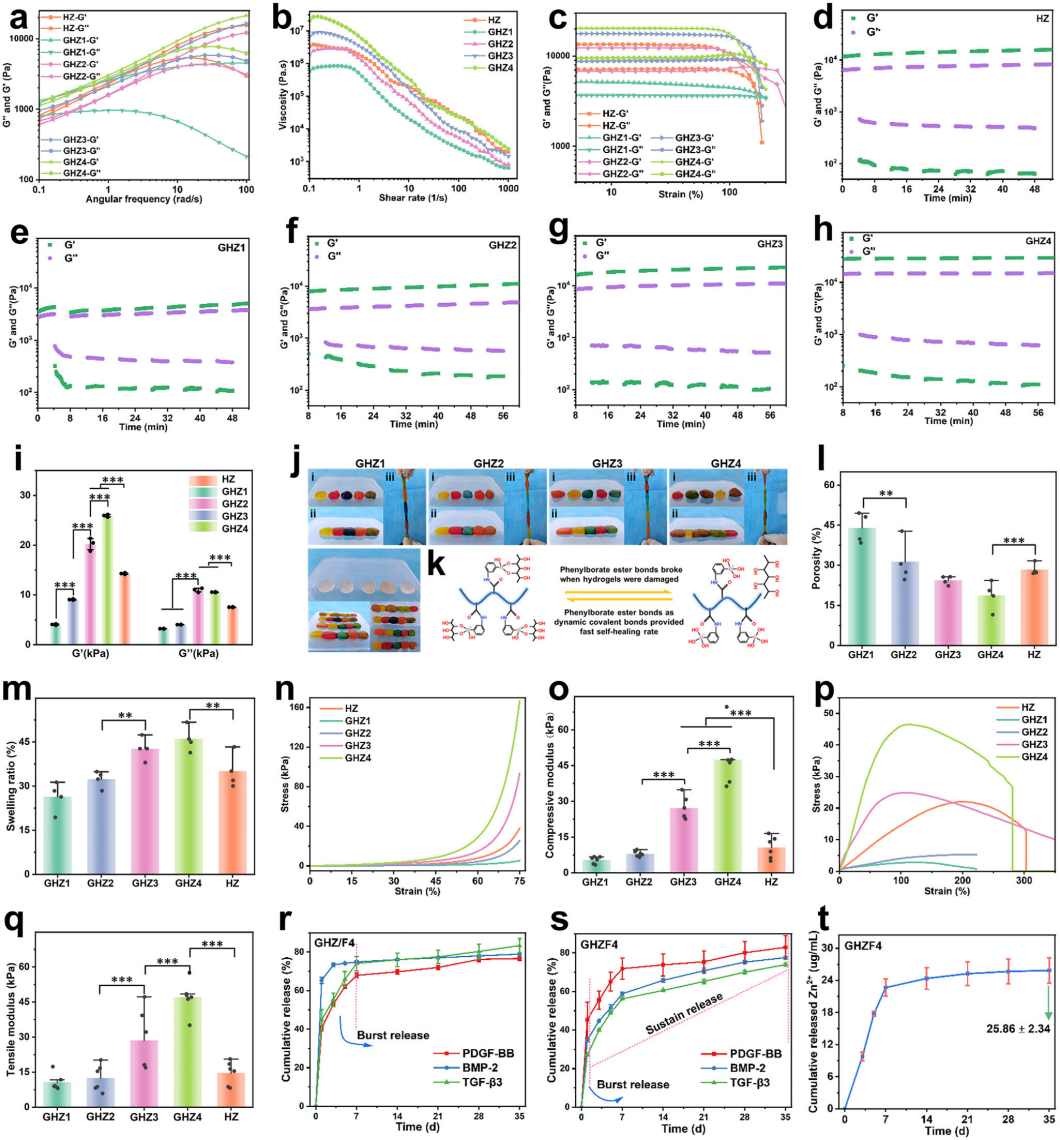
Characterization of the continuous gradient hydrogel. (a) Frequency scanning test of continuous gradient hydrogels. (b) The viscosity of the continuous gradient hydrogels. (c) Storage modulus (G′) and loss modulus (G″) of continuous gradient hydrogels measured via strain amplitude oscillation test (1 Hz, 25°C). (d–i) Variations in G’ and G” recorded from alternating step strain sweep tests of gradient hydrogel samples. (j) Digital photographs demonstrating the self‐healing performance of continuous gradient hydrogels. After 30 min of fusion, the reconnected hydrogel pieces sustain tensile stretching without fracture. (k) The self‐healing mechanism of hydrogels. (l) Porosity of continuous gradient hydrogel (*N* = 3). (m) Swelling ratio of continuous gradient hydrogel (*N* = 3). (n,o) Compressive stress‐strain curves, corresponding compressive modulus, and ultimate load of hydrogel specimens (*N* = 5). (p,q) Tensile stress‐strain curves, corresponding tensile modulus, and maximum load of hydrogels (*N* = 5). (r,s) In vitro cumulative release curves of PDGF‐BB, BMP‐2, and TGF‐β3 from GHZ/F4 (r) and GHZF4 (s) hydrogels in PBS at 37°C (*N* = 3). (t) In vitro cumulative Zn^2+^ release profiles of GHZF4 hydrogels. *p < 0.05, **p < 0.01, ***p < 0.001.

Every tested hydrogel shared comparable nonlinear mechanical rheology. The elevation of storage modulus (G’) and loss modulus (G″) as frequency increased represents a hallmark property of dynamically shape‐adaptable hydrogel matrices (Figure 2a) [32]. GHZ4 showed the highest G’ value, with the viscosity gradually increasing from GHZ1 to GHZ4 (Figure 2b). SBPS hydrogel displayed frequency‐dependent viscoelasticity contributing to its adhesive properties [32]: fluid‐like properties at low angular frequencies (0.1–2 rad s^−1^) for defect adaptation and solid‐like elasticity at high frequencies (2–100 rad s^−1^) for protection (Figure S7a,b). Lap‐shear measurement yielded an adhesive strength value of 6.93 ± 1.83 kPa (Figure S7c). Benefiting from abundant hydrogen bond interactions, the GHZF4 hydrogel displayed superior surface adhesion, matching the adhesive capacity of numerous tissue‐adhesive hydrogels reported in previous literature [33, 34, 35, 36, 37]. Strain sweeps confirmed solid‐like elasticity under critical shear strain, with GHZ4 transitioning between solid and fluid at 150% strain (Figure 2c). With a further increase of the strain to 200%, the G’ dramatically decreased due to the collapse of the hydrogel networks.

Self‐healing performance of the hydrogel was evaluated via alternating step strain scanning at a steady angular frequency (Figure 2d–h). Subjected to periodic switching between 200% large strain and 0.1% tiny strain, the hydrogel matrix suffered structural breakdown under high deformation. After strain reduction to 0.1%, both G′ and G″ swiftly recovered to their initial magnitudes, confirming fast reconstruction of the network upon multiple mechanical failures induced by large strain. The G′ and G″ of GHZ1 and GHZ4 gradually increased from 4077.18 ± 78.26 kPa to 25241.16 ± 3493.29 kPa, and 3229.95 ± 39.37 kPa to 12791.69 ± 2310.46 kPa, respectively (Figure 2i). Macroscopic testing showed that five stained hydrogel pieces merged into a single intact structure within 0.5 h, driven by dynamic functional group reactions (Figure 2j,k). GHZ4 hydrogels with intrinsic self‐healing and adaptive remodeling features maintain stable service duration even after structural breakage, making them durable tissue‐engineered constructs.

The porosity of HZ, GHZ1, GHZ2, GHZ3, and GHZ4 was 30.49 ± 6.15%, 43.68 ± 4.94%, 31.80 ± 7.01%, 24.52 ± 1.48%, and 18.71 ± 5.36%, respectively (Figure 2l). Similarly, the swelling ratio of HZ, GHZ1, GHZ2, GHZ3, and GHZ4 was 37.19 ± 10.07%, 26.35 ± 6.24%, 35.10 ± 7.19%, 42.71 ± 4.70%, and 46.03 ± 5.24%, respectively (Figure 2m). From GHZ1 to GHZ4, BSNF gradually aligns and accumulates under the electric field, densifying the network and reducing porosity. Meanwhile, the aligned nanofibers and enhanced cross‐linking form a stable framework that accommodates more water molecules, resulting in a gradual increase in swelling ratio. As shown in the strain‐stress curve of the compression test, the hydrogel's mechanical properties were measured in the direction perpendicular to the electric field and exhibited a gradient change (Figure 2n). Compressive modulus of GHZ1 and GHZ4 gradually increased from 5.35 ± 2.32 kPa to 47.53 ± 13.28 kPa (Figure 2o), and tensile modulus from 2.78 ± 0.69 kPa to 50.02 ± 13.14 kPa (Figure 2p–q), with maximum load showing similar trends (Figure S8a,b), confirming BSNF distribution's role in regulating the hydrogel gradients.

### 2.4 Characterization of Electric Field‐Pattern GFs‐Based Continuous Gradient Hydrogels

To achieve the spatial distribution of the bioactive factors, GFs‐based hydrogels were constructed via uniform incorporation of ZIF‐8/BMP‐2/8arm‐PEG‐NH_2_ and ZIF‐8/TGF‐β3/8arm‐PEG‐NH_2_ into PDGF‐BB‐preloaded hydrogel precursors. We evaluated the cumulative release behaviors of GFs from the GHZ/F4 and GHZF4 scaffolds.

Within GHZ/F4 hydrogel samples, the two bioactive factors exhibited rapid initial release throughout the entire incubation period (Figure 2r). By contrast, the GHZF4 system achieved a biphasic delivery mode: PDGF‐BB was rapidly released at the early stage, while BMP‐2 and TGF‐β3 were slowly released over a long time. This distinct release behavior originates from robust electrostatic bonding between the two growth factors and ZIF‐8 nanocarriers (Figure 2s). By day 35, cumulative BMP‐2 and TGF‐β3 release from GHZF4 was about 77.53 ± 0.77% and 73.94 ± 0.70%, respectively, compared to 74.93 ± 0.64% and 74.06 ± 3.70% released from GHZ/F4 within 7 days. ICP‐MS showed sustained Zn^2+^ release without a burst, reaching 25.86 ± 2.34 µg mL^−1^ by day 35 (Figure 2t).

### 2.5 Cytocompatibility and Recruitment Properties of Continuous Gradient Hydrogels for BMSCs In Vitro

**FIGURE 3:**
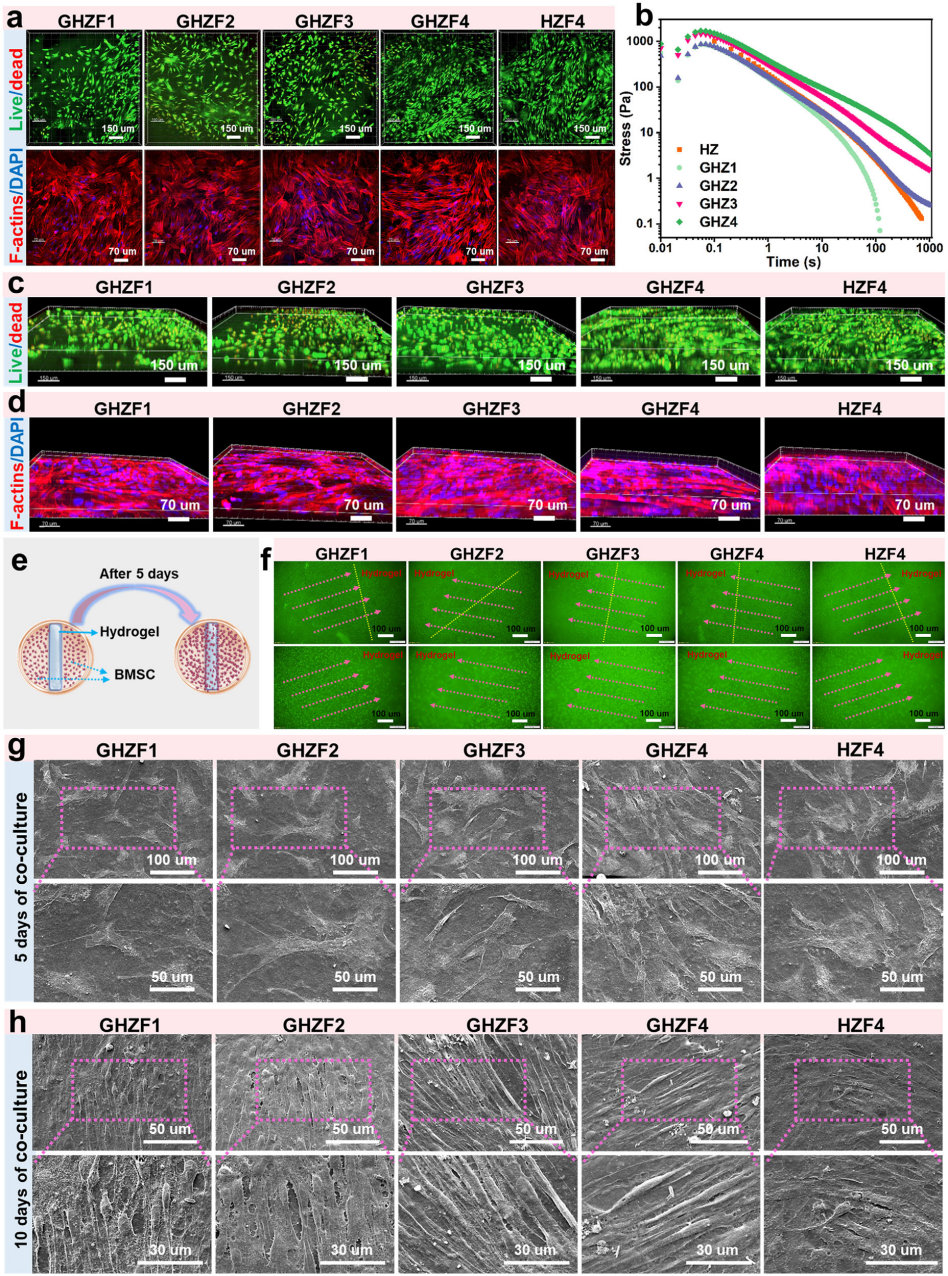
Cell recruitment performance of hydrogels in vitro. (a) Cell adhesion on the hydrogel surface. (b) Stress relaxation of the hydrogels. (c) CLSM captures 3D fluorescence images to evaluate cell migration within the hydrogel. (d) F‐actin/DAPI staining showing the cell migration in the hydrogel by CLSM. (e,f) Schematic illustration of the BMSCs migration assay in vitro, and Calcein‐AM/PI staining. (g,h) The SEM images showing the morphology of cells cultured onto the hydrogel after 5 and 10 days, respectively.

Calcein‐AM/PI staining and CCK8 assay confirmed >95% BMSCs viability and normal proliferation in hydrogel extracts (Figures S9 and S10). BSMCs exhibited similar cell spreading and viability across hydrogel surfaces, verifying good cytocompatibility (Figure 3a).

Successful homing of mesenchymal stem cells represents the initial pivotal stage of biomaterial‐guided in‐situ tissue repair. Well‐documented evidence confirms that fully interconnected pore architectures facilitate sufficient diffusion of oxygen and nutritional substances, creating favorable conditions for cell homing and subsequent tissue penetration into scaffold interiors. Beyond that, the shape‐adaptable hydrogel with reversible cross‐link domains allows structural rearrangement under mechanical traction exerted by cells, providing a favorable microenvironment for cellular movement across the hydrogel interior network. Viscoelasticity and inherent stress relaxation represent an intrinsic mechanical property of natural ECM [15]. Hence, the capacity to undergo stress relaxation becomes a core mechanical property enabling hydrogels to recapitulate physiological cell‐matrix communications in primary fracture hematomas. Stress relaxation tests showed >99% stress dissipation within 1000 s under 15% strain, enabling cell‐mediated matrix remodeling (Figure 3b). CLSM and F‐actin/DAPI staining demonstrated enhanced BMSC recruitment and migration toward GHZF4 compared to other groups (Figure 3c,d). All results in Figure 3a–f were obtained via direct cell‐hydrogel co‐culture, which intuitively reflects cell adhesion on the hydrogel surface and directional migration toward and infiltration into the hydrogel scaffold. To further compare the cell homing capacity of distinct hydrogel groups, uniform‐width and uniform‐thickness hydrogel strips were pre‐placed at the bottom of each culture well at the 0 h time point, followed by cell seeding on two lateral sides of the hydrogel constructs (Figure 3e). After incubation at 37°C for 5 days, Calcein‐AM/PI staining results demonstrated that BSMCs co‐cultured with various hydrogel specimens displayed comparable migratory tendencies toward hydrogel regions, indicating that all hydrogels could facilitate BMSCs migration (Figure 3f). The cell recruitment effect in the GHZF4 group was superior to that of the GHZF1, GHZF2, and GHZF3 groups, attributed to the multiple gradient signals and enhanced mechanical strength.

### 2.6 Effect of the Orientation Gradient of the Hydrogel on Cell Behavior

Consistent with our anticipation, the directional gradient structure within hydrogels exerts regulatory effects on cellular adhesion and aggregation phenotypes. Following 5 days of in vitro cultivation, cells grown on HZF4, GHZF1, GHZF2, and GHZF3 substrates presented elongated spindles and polygonal morphologies, but showed relatively oriented structures on GHZF4 (Figure 3g). After being cultured on the hydrogels for 10 days, cells grew along aligned GHZF4, exhibiting better cytocompatibility than non‐aligned HZF4 (Figure 3h). The hydrogel surface was partially covered by compact interconnected cell layers, with noticeable protrusions indicating effective cellular infiltration into the hydrogel interior. The hydrogels exhibited a high degree of cell spreading and extended lamellipodia formation over the hydrogel surface, indicating that the hydrogel could support cell adhesion, infiltration, and proliferation. Numerous published works have proven that patterned microarchitectures from different biomaterials serve as critical regulators of cell fate, covering cell polarization, proliferation, and osteogenic differentiation [38].

### 2.7 Recruitment and Angiogenesis of Continuous Gradient Hydrogels In Vitro

**FIGURE 4:**
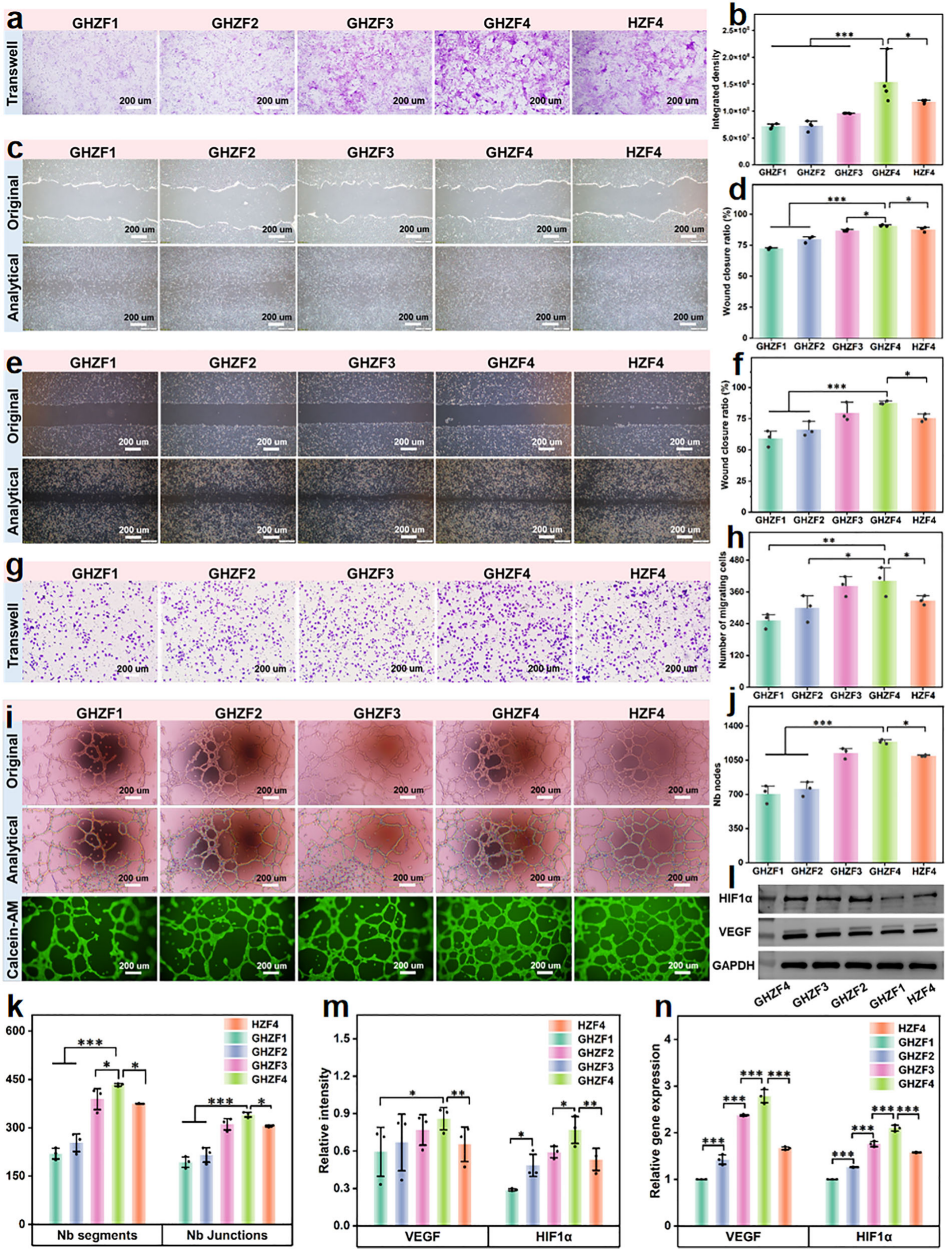
Recruitment and angiogenesis performance of hydrogels in vitro. (a) Transwell assay evaluated the migration activity of BMSCs cultured with hydrogel, where the migrated cells were stained with crystal violet. (b) The corresponding quantitative analysis. (c,d) Scratch assay and corresponding quantitative analysis (BMSCs). (e,f) Scratch assay and corresponding quantitative analysis (HUVECs). (g,h) Crystal violet staining and corresponding quantitative analysis. (i) Optical microscope images of the Matrigel experiment. (j,k) Quantitative analysis of the tube formation ability. (l) Angiogenic protein expression was evaluated by Western blot. (m) Quantitative analysis of angiogenic protein expression of HUVECs. (n) Angiogenic gene expression was evaluated by RT‐qPCR. *p < 0.05, **p < 0.01, ***p < 0.001.

Scratch wound healing, transwell migration, and tubular formation tests were conducted with BMSCs and HUVECs to evaluate the in vitro angiogenic capacity of the hydrogel matrices. All assays in Figure 4 were conducted using hydrogel extracts to avoid physical obstruction of the hydrogel scaffold, which could interfere with staining observation and quantitative analysis. Transwell assays showed HZF4 and GHZF4 groups had higher BMSC migration than other hydrogels after 24 h (Figure 4a,b). Scratch assays demonstrated GHZF4 accelerated BMSC wound closure (Figure 4c,d) and HUVEC migration (10.7‐fold higher than GHZF1) (Figure 4e,f). Transwell assays confirmed more migrated HUVECs in HZF4 and GHZF4 groups (Figure 4g,h). Matrigel experiment (tube formation assay) showed increased tube structures in HZF4, GHZF3, and GHZF4, with GHZF4 exhibiting 1.97‐fold more segments and 1.76‐fold more junction numbers in the GHZF4 group compared with the GHZF1 group (Figure 4i–k and Figure S11). Western blot and RT‐qPCR revealed that GHZF4 upregulated HIF‐1α and VEGF expression (2.09‐fold for HIF‐1α gene and 2.78‐fold for VEGF gene, 2.66‐fold for HIF‐1α protein and 1.45‐fold for VEGF protein) compared to HZF4 (Figure 4l–n). The significantly enhanced bioactivity of GHZF4 is attributed to three key factors: (1) burst release of PDGF‐BB efficiently recruits BMSCs and vascular endothelial cells; (2) gradient structure and mechanical cues provide favorable guidance for cell migration; (3) sustained release of Zn^2+^ exerts immunomodulatory and pro‐angiogenic effects.

### 2.8 Spatiotemporally Releasing GFs Induced Stem Cell Differentiation In Vitro

**FIGURE 5:**
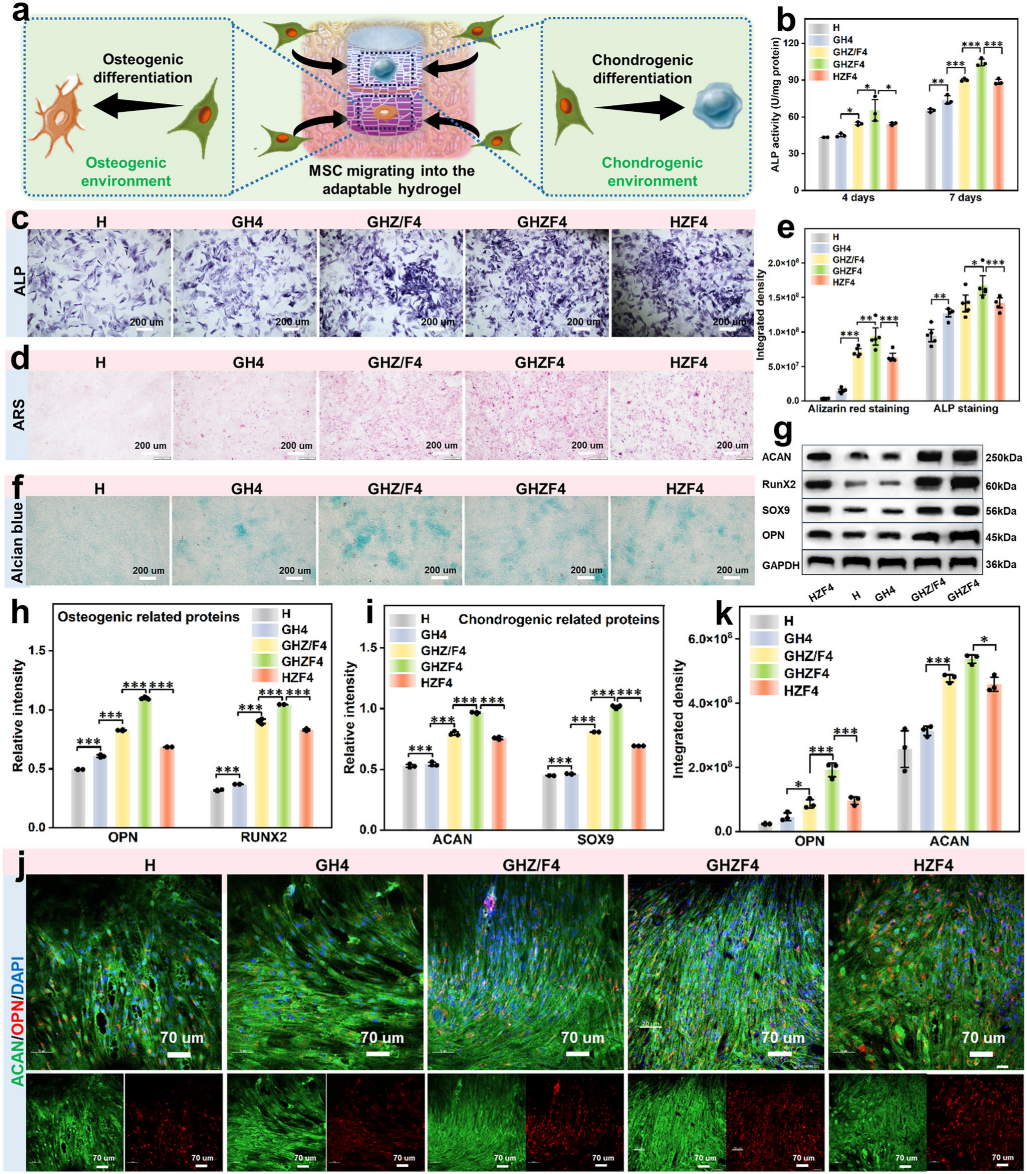
Chondrogenic and osteogenic differentiation mediated by gradient hydrogels. (a) Schematic diagram illustrating hydrogel‐guided dual‐lineage differentiation toward cartilage and bone. (b) Quantitative detection of ALP activity. (c) ALP staining of BMSCs seeded on hydrogel matrices. (d) Alizarin Red S (ARS) staining of BMSCs. (e) ImageJ‐based quantitative statistics for ALP and ARS staining intensity. (f) Alcian blue staining for chondrogenic extracellular matrix deposition. (g) Western blot bands of osteogenic markers (OPN, RUNX2) and chondrogenic markers (SOX9, ACAN) from BMSCs cultured with hydrogels. (h,i) Quantitative analysis results of osteogenic and chondrogenic protein levels via ImageJ. (j) CLSM micrographs of ACAN (chondrogenic) and OPN (osteogenic) immunofluorescence in BMSCs after 21 days of hydrogel culture. (k) Corresponding ImageJ quantification of immunofluorescence intensity. *p < 0.05, **p < 0.01, ***p < 0.001.

To validate the hydrogel's regulatory effects on cellular phenotypes, we further investigated its potency to trigger chondrogenic and osteogenic lineage differentiation of stem cells. Under physiological conditions, endogenous stem cells migrate toward injury sites, receive biochemical cues from the adjacent extracellular microenvironment, and subsequently differentiate into multiple specialized cell populations. Such sequential biological events ultimately facilitate the reconstruction of impaired ECM and drive tissue regenerative remodeling (Figure 5a). Alkaline phosphatase (ALP) activity assays showed that BMSCs on GHZF4 had much higher activity at 7 and 14 days (Figure 5b). Following 14 and 28 days of incubation within osteochondral induction medium, ALP and Alizarin Red S (ARS) staining were separately applied to visualize extracellular collagen secretion and calcium mineral deposition from BMSCs. The corresponding micrographs showed significantly more pronounced coloration in GHZF4, GHZ/F4, and HZF4 groups compared with other groups, with GHZF showing the highest activity (Figure 5c,d). Quantitative analysis revealed that the GHZF4 group exhibited a 26.74‐fold higher absorbance value for ARS staining and 1.76‐fold elevated ALP enzymatic activity relative to the H group (Figure 5e). Alcian blue staining (14 days) confirmed that GHZF4 had the highest glycosaminoglycan (GAG) content (Figure 5f). Western blot (Figure 5g) and corresponding quantitative analysis revealed significantly upregulated osteogenic and chondrogenic protein markers in the GHZF4 group relative to the H group. For osteogenic indicators, osteopontin (OPN) and Runt‐related transcription factor 2 (RUNX2) exhibited 2.24‐fold and 3.28‐fold higher expression levels, respectively (Figure 5h). Meanwhile, the chondrogenic biomarkers SRY‐Box Transcription Factor 9 (SOX9) and aggrecan (ACAN) were elevated by 2.24‐fold and 1.83‐fold, respectively (Figure 5i). Immunofluorescent staining (21 days) confirmed the highest OPN and ACAN expressions in GHZF4, followed by HZF4≈GHZ/F4, GH4, and H groups (Figure 5j,k). These collective data verify that the GHZF4 possesses superior capacity to facilitate osteochondral lineage differentiation of stem cells.

### 2.9 In Vitro Anti‐Oxidation, Anti‐Inflammation, and Bone Regulation of GHZF4

**FIGURE 6:**
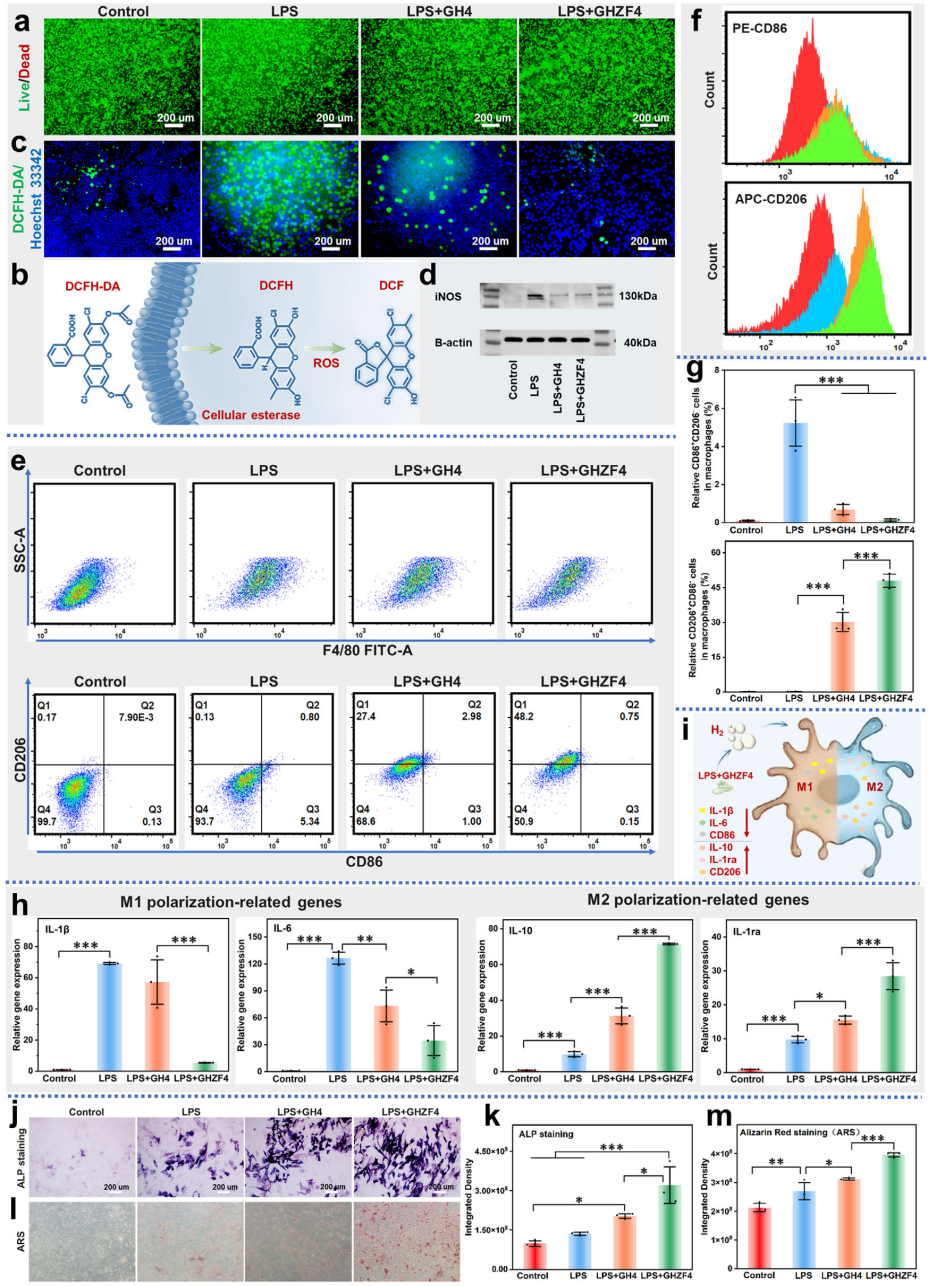
In vitro anti‐oxidation, anti‐inflammation, and bone regulation of GHZF4. (a) Live/dead fluorescence staining of LPS‐stimulated RAW264.7 macrophages under distinct hydrogel interventions. (b) Schematic diagram of intracellular ROS detection mechanism using DCFH‐DA fluorescent probe. (c) CLSM micrographs of DCFH‐DA‐labeled RAW264.7 cells across treatment groups. (d) Western blot detection of M1 macrophage marker iNOS in RAW264.7 cells. (e) Flow cytometry profiling of RAW264.7 cells stained with CD86 (M1) and CD206 (M2) antibodies. (f,g) Corresponding quantitative statistics of flow cytometry results. (h) RT‐qPCR quantification of M1 genes (IL‐1β, IL‐6) and M2 genes (IL‐1ra, IL‐10) in treated RAW264.7 cells. (i) Schematic showing GHZF4‐mediated M1‐to‐M2 macrophage phenotypic repolarization. (j) ALP staining of MC3T3‐E1 cells with varied treatments. (k) Quantitative analysis of ALP staining results. (l) Alizarin Red S (ARS) staining of MC3T3‐E1 cells. (m) Corresponding quantification of ARS staining intensity. *p < 0.05, **p < 0.01, ***p < 0.001.

Implantable biomaterials with favorable immune responses are critical for guiding successful osteogenesis and angiogenesis [39, 40]. Consequently, we evaluated the immunomodulatory properties of the GHZF4 hydrogel. Macrophage biocompatibility was assessed using live/dead staining following 48 h incubation with lipopolysaccharide (LPS), LPS+GH4, or LPS+GHZF4. Negligible cytotoxicity was observed across all treatment groups (Figure 6a). DCFH‐DA staining confirmed that GHZF4 attenuated LPS‐induced intracellular ROS more effectively than GH4 (Figure 6b,c), attributed to ROS‐sensitive borate bonds within the hydrogel network and the sustained release of Zn^2+^ [41, 42]. Western blot showed GHZF4 downregulated pro‐inflammatory iNOS expression (Figure 6d). Flow cytometry analysis revealed that LPS promoted M1 phenotype (elevated CD86), while GHZF4 significantly upregulated M2‐associated marker CD206 (Figure 6e–g). RT‐qPCR detection verified that the GHZF4 hydrogel downregulated pro‐inflammatory mediators IL‐1β and IL‐6, while elevating the expression of anti‐inflammatory factors IL‐1ra and IL‐10 (Figure 6h), driving M1‐to‐M2 macrophage polarization (Figure 6i). This phenotypic shift is crucial for inflammation resolution and is critically dependent on the restoration of oxidative homeostasis within M1 macrophages [43].

It is widely acknowledged that anti‐inflammatory cytokines, especially M2 macrophage‐derived IL‐10, can promote stem cell osteogenic differentiation [44]. Following 7 days of culture, MC3T3‐E1 cultured in conditioned medium (derived from supernatant of macrophages treated with LPS+GHZF4) exhibited significantly enhanced ALP expression compared to other groups (Figure 6j,k). ARS staining performed on day 21 revealed the most intense mineralization in cultures treated with LPS+GHZF4‐conditioned medium (Figure 6l,m). The significantly increased ALP activity and mineralized nodule deposition in the LPS+GHZF4 group suggest that oxidative stress impairs osteogenic differentiation, while GHZF4 supplementation enhances osteogenesis through antioxidative mechanisms and promotion of bone mineralization. Beyond modulating polarization, the immunoregulatory microenvironment established by implantable biomaterials also stimulated extensive filopodia formation in macrophages in vitro and enhanced the secretion of endogenous pro‐regenerative factors VEGF and BMP‐2 by M2‐polarized macrophages [45].

### 2.10 Osteochondral Reconstruction by Hydrogel in a Rat Model

**FIGURE 7:**
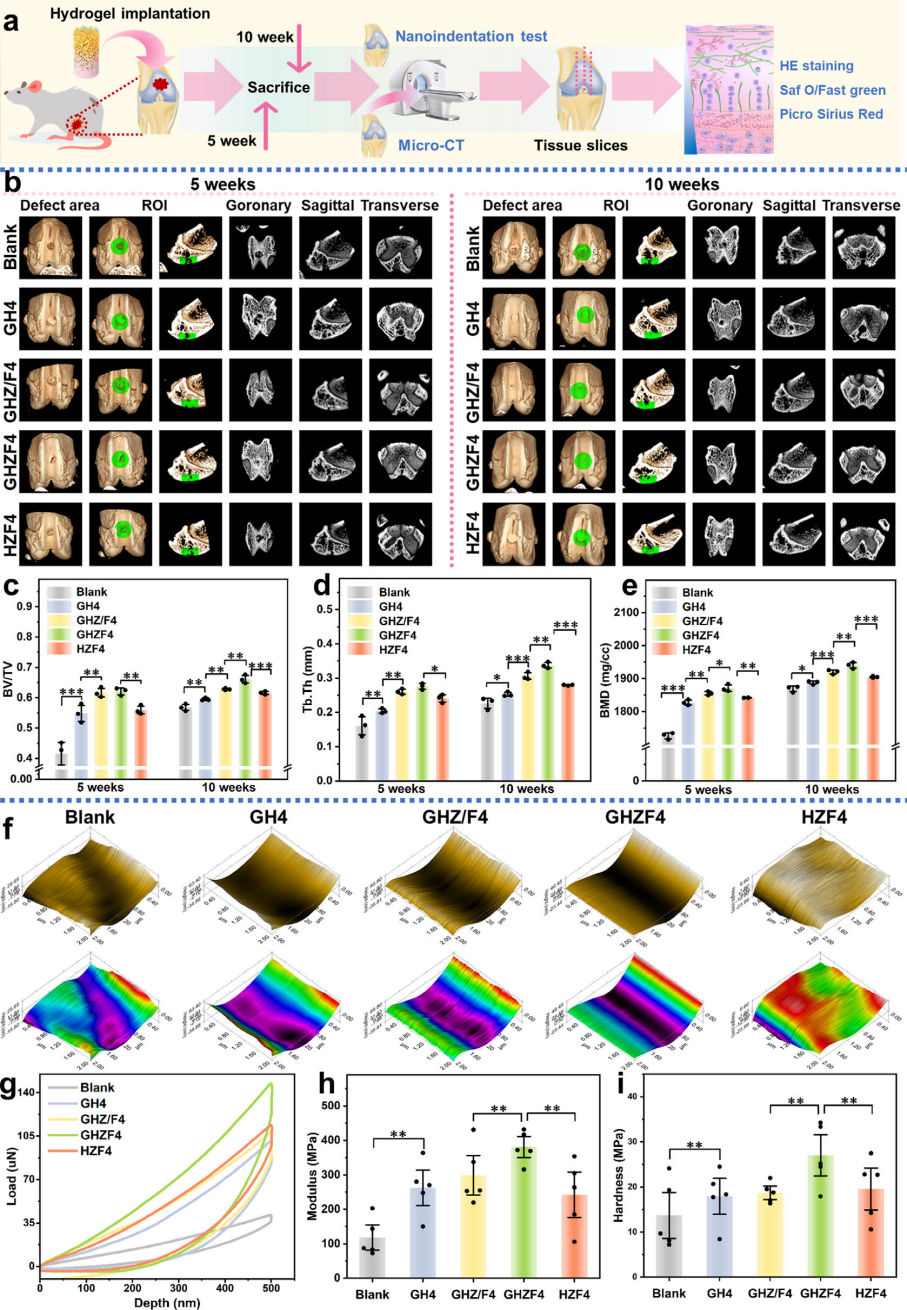
Osteochondral defects were repaired by different hydrogels in the rat knee joint model at 5/10 weeks post‐surgery. (a) Schematic illustration of surgical procedures for rat knee osteochondral defect model. (b) 3D reconstructed micro‐CT images (green circles mark regenerated subchondral bone). (c–e) Quantitative micro‐CT analysis of bone parameters: (c) bone volume/total volume (BV/TV), (d) trabecular thickness (Tb. Th), (e) bone mineral density (BMD). (f) Surface topography of repaired tissues captured via nanoindentation measurement. (g) Typical load‐displacement curves of regenerated tissues from different groups. (h,i) Statistical comparison of repaired tissue biomechanics including reduced modulus (h) and hardness (i). *p < 0.05, **p < 0.01, ***p < 0.001.

Blank scaffold, HZF4, GH4, GHZ/F4, and GHZF4 hydrogels were implanted to repair osteochondral defects in rat knee joints (Figure 7a). Hematoxylin‐eosin (HE) staining of major visceral organs harvested at week 2 validated the favorable in vivo biocompatibility of these hydrogels (Figure S12). Micro‐CT scanning was performed to assess newly regenerated osteochondral tissue at 5 and 10 weeks after implantation (Figure 7b and Figures S13 and S14). Micro‐CT showed the Blank group had residual cavities at 5 weeks, while other groups exhibited peripheral new bone formation with gaps; GHZF4 showed obvious but non‐uniform regeneration. At 10 weeks, GHZF4‐treated defects were filled with smooth, well‐integrated tissue, outperforming other groups. Quantitative analysis further demonstrated significantly elevated bone volume fraction (BV/TV), trabecular thickness (Tb.Th), and bone mineral density (BMD) within the trabecular region of interest for the GHZF4 group relative to all other experimental groups (Figure 7c–e and Figure S15). Collectively, GHZF4 yielded the optimal osteochondral regenerative capacity, with GHZ/F4, HZF4 and GH4 groups exhibiting progressively inferior repair effects in sequence.

To evaluate the biomechanical properties of regenerated osteochondral tissue, nanoindentation testing was performed to assess whether the neotissue could withstand the complex mechanical environment of the joint. At 10 weeks post‐implantation, the GHZF4 group exhibited significantly smoother articular surfaces compared to the irregular, roughened surfaces observed in the blank control group (Figure 7f). Load‐displacement curves further demonstrated that the mechanical behavior of the HZF4 and GHZ/F4 groups approached that of GHZF4, while GH4 displayed inferior performance (Figure 7g). Mechanical quantification illustrated that the regenerated tissue from the GHZF4 group possessed superior hardness relative to all remaining experimental groups (Figure 7i ). Consistently, the reduced modulus followed the same trend, with GHZF4 achieving the highest value, significantly surpassing GHZ/F4, GH4, HZF4, and Blank (Figure 7h). Notably, although the GH4, HZF4, and GHZ/F4 groups all showed significantly enhanced biomechanical properties versus the Blank group (*P*<0.05), the combined action of structural gradation and spatiotemporal delivery collectively promotes biomimetic osteochondral repair.

**FIGURE 8:**
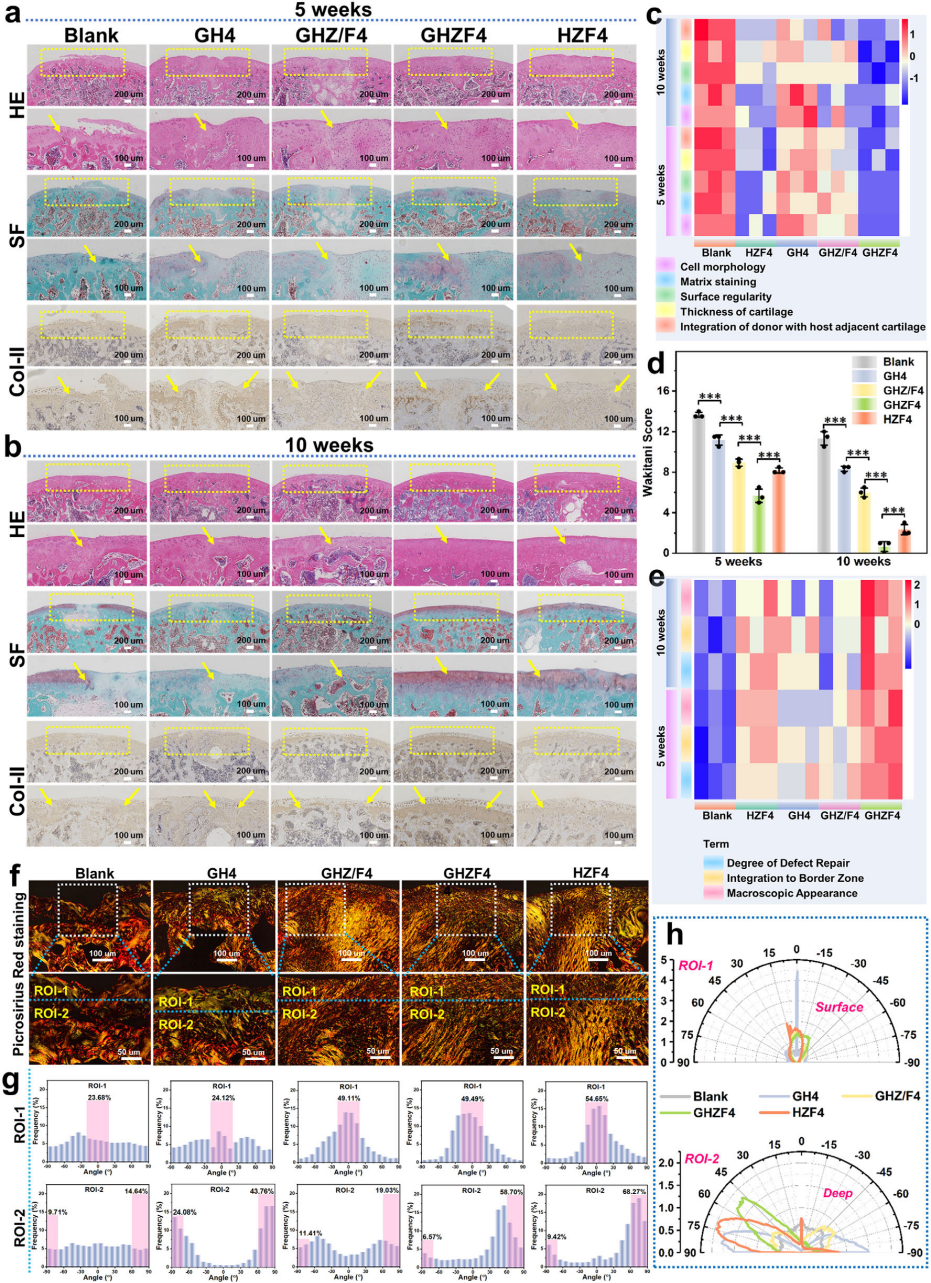
Histological staining of in vivo osteochondral restoration at 5 and 10 weeks following hydrogel implantation. (a,b) Full‐view and magnified micrographs of H&E and Safranin O/Fast Green stained tissues harvested at 5 and 10 weeks postoperatively (dotted rectangles mark the defect regions). (c,d) Quantitative histological grading via modified Wakitani scoring system, covering cartilage structure, cellular irregularity and matrix staining intensity. (e) Macroscopic repair scores assessed by the International Cartilage Repair Society (ICRS) classification standard. (f) Picrosirius Red‐stained tissue slices at week 10 captured under polarized light microscopy (magnified views of the framed area in the upper panel are displayed below). (g) Corresponding quantitative results of Picrosirius Red‐stained tissue slices for analyzing collagen fiber alignment and dispersion within lesions. (h) Polar coordinate statistical analysis quantifying the angular distribution of neo‐collagen fibrils across groups. Fibers parallel to the joint surface correspond to 0°, while vertically oriented fibrils perpendicular to the surface register at 90°. *p < 0.05, **p < 0.01, ***p < 0.001.

We further assessed the in vivo repair efficacy of osteochondral lesions through histological staining and immuno‐histochemical detection. Histological staining verified that GHZF4 remarkably boosted simultaneous regeneration of cartilage and subchondral bone compared with other cohorts (Figure 8a,b). 5 weeks after implantation, blank control groups displayed large hollow cavities and obvious interfacial fissures, along with degenerative collapse of native surrounding cartilage. By contrast, all hydrogel‐treated groups only achieved partial bone restoration, with nascent bone tissues predominantly accumulating along defect margins. Massive endogenous cells infiltrated and colonized the implanted GHZF4 scaffold inside lesion sites, which reveals the hydrogel acts as a bioactive template to orchestrate bone regrowth and promote stable integration between osseous tissue and the self‐adaptive matrix (Figure 8a). At the 10‐week time point, GHZF4‐treated defects developed a continuous, even neo‐cartilage layer whose thickness matched intact peripheral cartilage. The junction between regenerated and host cartilage fully fused, and chondrocytes within newly formed tissue arranged in a pattern characteristic of native hyaline cartilage. For GH, HZF4, and GHZ/F4 groups, cartilage reconstruction remained incomplete despite tight bonding between neo‐cartilage and subchondral bone (Figure 8b). The blank group merely exhibited minimal osteochondral regrowth after 10 weeks of healing, demonstrating poor repair quality.

Immunohistochemical staining showed higher chondrocyte and COL‐II expression in GHZF4 (Figures S16 and S17). We utilized Wakitani's histological scoring system to quantify the extent of cartilage repair across different groups, with smaller scores corresponding to superior repair capacity (Figure 8c,d), following the criteria outlined in Table S2. At both the 5‐ and 10‐week time points, GHZF4 attained the minimal scores relative to all other treatment groups. Furthermore, macroscopic assessment based on the International Cartilage Repair Society (ICRS) grading system (Table S3) yielded peak values for GHZF4: 5.17 ± 1.15 at week 5 and 10.5 ± 0.87 at week 10 after implantation (Figure 8e and Figure S18).

### 2.11 Characterization of Hierarchical Anisotropic Architectures in Regenerated Osteochondral Extracellular Matrix

Type II collagen dominates native hyaline articular cartilage, while fibrocartilage co‐expresses type I and II collagens, and fibrous tissue solely secretes type I collagen without type II deposition. Accordingly, collagen II immunoreactivity serves as an indicator of hyaline chondrogenic maturation, yet single collagen II staining fails to accurately identify genuine hyaline cartilage, given its coexistence within fibrocartilage matrices. Authentic hyaline cartilage regeneration can only be validated by three combined features: abundant collagen II expression, negligible or undetectable collagen I signals, and well‐aligned collagen fibrillar networks observable under polarized microscopy. Sirius Red staining coupled with polarizing light microscopy was implemented to visualize the directional arrangement of collagen fibrils (Figure 8f). The dominant orientation angle of collagen fibers was quantified with the Directionality plugin embedded in ImageJ. Referencing the layered structure of intact osteochondral tissue, two separate regions of interest (ROI) were defined for quantitative comparison: the superficial zone occupying the top one‐third of the cartilage layer, and the middle‐deep zone covering the bottom two‐thirds of cartilage tissue (Figure 8g,h). Sirius red staining and polarizing microscopy showed native articular cartilage has horizontal surface collagen fibers and vertical middle/deep fibers. Blank group had random collagen orientation, while HZF4 showed 67.84% vertical fibers (fibrous connective tissue). GH4 had 49.11% horizontal surface fibers and 30.44% vertical deep fibers (random fibrous tissue). GHZ/F4 had 49.49% parallel surface fibers and 65.27% vertical fibers. GHZF4 achieved 54.65% parallel surface fibers and 77.69% vertical deep fibers, closest to native tissue (≈90% horizontal surface, ≈75% vertical deep), confirming the continuous gradient's role in reconstructing anisotropic ECM.

### 2.12 Transcriptomic Profiling of Regenerated Osteochondral Tissues

**FIGURE 9:**
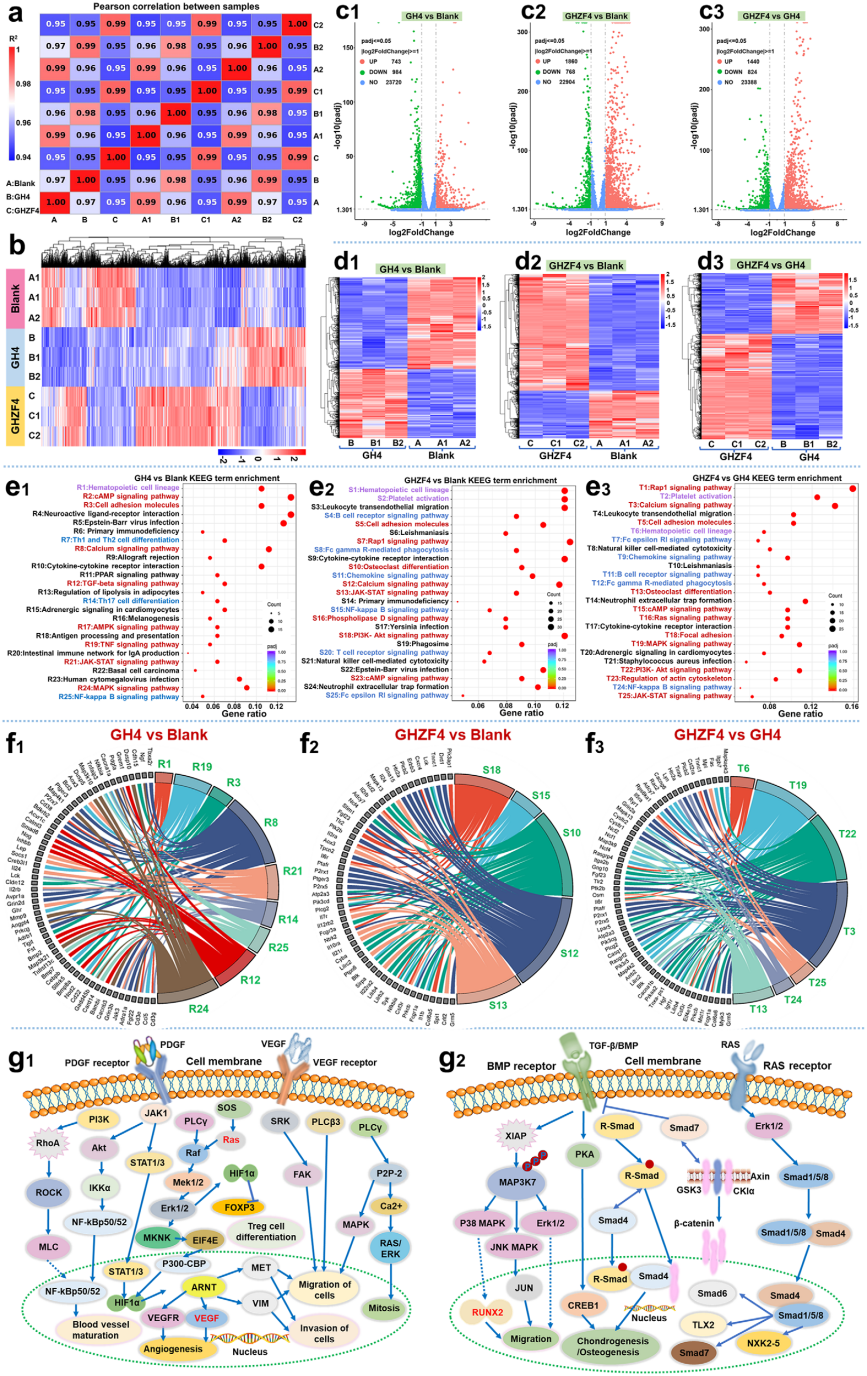
Transcriptomic profiling of regenerated osteochondral tissue uncovering hydrogel‐mediated regenerative molecular pathways. (a) Pearson correlation heatmap across all samples. (b) Volcano plots displaying transcriptomic differentially expressed genes (DEGs), *N* = 3 biological replicates per group. (c) The heatmap of the differentially expressed genes identified by transcriptome sequencing. (d) Pairwise comparative heatmaps of DEGs from distinct treatment groups. (e) Enriched upregulated KEGG pathways associated with cell behavior, osteogenic differentiation, angiogenesis, cell recruitment and inflammatory modulation. (f) Circular visualization illustrating gene‐pathway interactions of the selected upregulated KEGG terms. (g) Core canonical signaling pathways enriched by DEGs, analyzed via transcriptome sequencing combined with IPA software.

Transcriptome sequencing was conducted on repaired osteochondral tissues harvested from Blank, GH4 and GHZF4 to explore the molecular mechanisms by which GHZF4 hydrogel facilitates osteochondral regeneration. Box plots (Figure S19a), principal component analysis (PCA) (Figure S19b), and Pearson correlation heatmap (Figure 9a) verified the robust biological repeatability of each group and and prominent transcriptional disparities across different treatments. The heatmap of differentially expressed genes (DEGs) showed distinct expression profiles between groups, with active genes marked in red and silenced genes in blue (Figure 9b). Venn diagram analysis revealed 1727 DEGs in GH4 vs Blank, 2628 DEGs in GHZF4 vs Blank, and 2264 DEGs in GHZF4 vs GH4, with 11899 genes co‐expressed in all groups (Figure S20). Volcano plots visualized 743 up‐regulated and 984 down‐regulated genes in GH4 vs Blank, 1860 up‐regulated and 768 down‐regulated genes in GHZF4 vs Blank, and 1440 up‐regulated and 824 down‐regulated genes in GHZF4 vs GH4 (Figure 9c1–c3). Hierarchical clustering heatmaps further corroborated substantial discrepancies in gene expression patterns across all pairwise comparisons (Figure 9d1–d3).

Functional enrichment analyses including Gene Ontology (GO) and Kyoto Encyclopedia of Genes and Genomes (KEGG) were performed to interpret the biological roles of these DEGs. The top 30 enriched GO terms and chord diagrams of up‐regulated GO pathways in each comparison group were shown in Figures S21–S26. GO functional enrichment demonstrated that differential transcripts specific in the GHZF4 group were primarily involved in secretory granules, immune response regulation, and cytokine receptor activity. KEGG pathway enrichment further uncovered that altered genes within the GH4 group accumulate in signaling cascades controlling immunoregulation, stem cell development and osteogenic differentiation.

Notably, transcriptomic analysis suggested that GHZF4 treatment potentially up‐regulated pathways related to leukocyte migration (cell recruitment), immunomodulation (NF‐κB/FcεRI), angiogenesis and osteochondral differentiation (PI3K‐Akt/JAK‐STAT) (Figure 9e–g). These transcriptomic findings provide important potential mechanistic clues for understanding the orchestrated effects of GHZF4 hydrogel on immunomodulation, stem cell recruitment, angiogenesis, and osteochondral differentiation, and also offer key directions for further in‐depth mechanistic research in future studies. Collectively, the transcriptomic analysis supports that the multilevel gradient cues and bioactive factor release of GHZF4 hydrogel can regulate the gene expression related to the core processes of osteochondral regeneration, thereby remodeling the regenerative microenvironment and promoting functional osteochondral repair.

### 2.13 Osteochondral Reconstruction in the Rabbit Model

**FIGURE 10:**
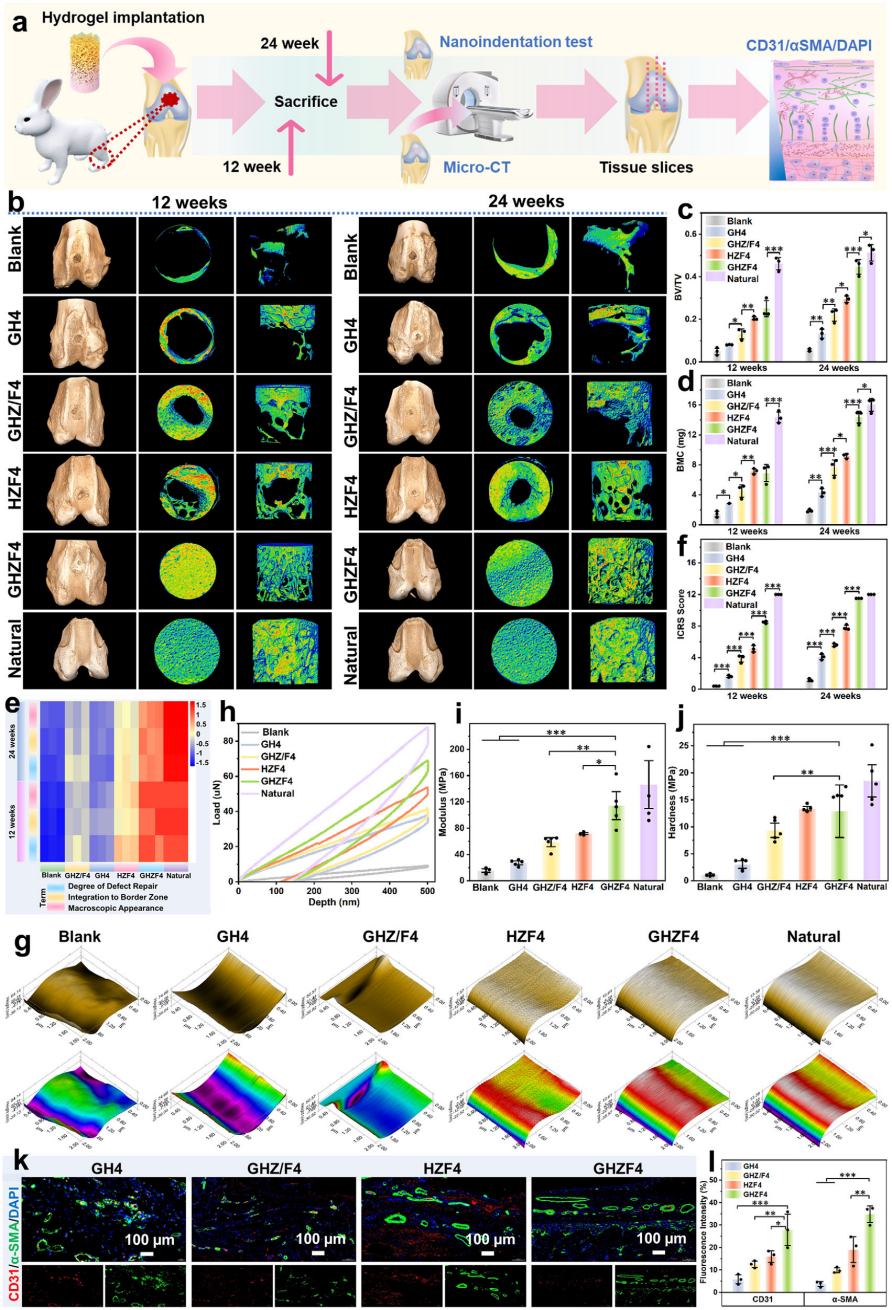
Hydrogel‐promoted in situ osteochondral regeneration in a rabbit knee defect model. (a) Schematic of the experimental protocol for rabbit knee osteochondral repair. (b) 3D‐reconstructed micro‐CT images at 12 and 24 weeks, green circles mark newly formed subchondral bone. (c,d) Quantitative micro‐CT assessment of repaired samples, including BV/TV (c) and BMD (d). (e,f) ICRS macroscopic scoring and quantitative analysis of gross repair performance. (g) Surface topography of repaired tissues obtained via nanoindentation testing. (h) Representative load‐displacement curves of repaired tissues for each group. (i,j) Biomechanical properties of repaired tissues, including reduced modulus (i) and hardness (j). (k,l) CLSM images and corresponding ImageJ‐quantified fluorescence intensity of CD31 and α‐SMA in repaired osteochondral tissue. *p < 0.05, **p < 0.01, ***p < 0.001.

A Schematic diagram (Figure 10a) illustrating the experimental design in rabbit knee joint, including the establishment of full‐thickness osteochondral defects in the femoral condyle, local implantation of different hydrogel groups (GHZF4, HZF4, GHZ/F4, GH4) or no treatment (Blank group). Systematic evaluations were conducted at 12 and 24 weeks after surgery, with intact native knee joint tissue used as the positive control (Natural group).

3D‐reconstructed micro‐CT images of defect sites at 12 and 24 weeks after surgery (Green circles highlight the newly formed subchondral bone) (Figure 10b and Figure S27). The GHZF4 group exhibited the most abundant continuous new subchondral bone at 12/24 weeks, with a morphology closest to the Natural group at 24 weeks post‐treatment. The HZF4 group showed slightly less new bone formation than GHZF4, followed by the GHZ/F4 and GH4 groups. The Blank group only presented sparse, discontinuous new bone. Quantitative micro‐CT assessment of subchondral bone mass within repaired specimens revealed that BV/TV and BMD values in the GHZF4 group were markedly elevated relative to all other experimental cohorts (*p* < 0.05), while no statistically significant discrepancy was observed compared with the Natural group (*p* > 0.05) (Figure 10c,d), confirming its superior ability to promote subchondral bone regeneration. ICRS macroscopic scores demonstrated that the GHZF4 group obtained the highest ICRS scores, which were close to those of the Natural group (Figure 10e). The scoring order among other groups was HZF4> GHZ/F4> GH4> Blank, where the Blank group showed the lowest scores due to incomplete defect filling and rough cartilage surface (Figure 10f). These quantitative data confirmed that the GHZF4 hydrogel most effectively improved the macroscopic integrity of the repaired cartilage.

Biomechanical properties of the repaired tissues were quantitatively characterized via nanoindentation tests. The GHZF4 group exhibited a smooth, uniform surface morphology highly similar to the Natural group's normal cartilage. The HZF4 group showed mild surface irregularities, while the GHZ/F4 and GH4 groups had increased surface roughness (Figure 10g). The blank group showed severe unevenness with exposed subchondral bone. Representative load‐displacement loops of repaired tissues (Figure 10h) showed that the GHZF4 group's load‐displacement loop was closest to the Natural group, with a narrow hysteresis loop and stable load‐bearing capacity. The HZF4 group showed a slightly wider loop, GHZ/F4 and GH4 groups had obvious loop expansion and reduced load‐bearing stability, while the Blank group had an irregular loop. The GHZF4 group had the highest reduced modulus (114.27 ± 31.76 MPa) and hardness (15.50 ± 7.25 MPa), which showed no significant difference compared with the Natural group (146.27 ± 54.46 MPa, 20.33 ± 4.45 MPa, *p* > 0.05). This quantitatively confirms that GHZF4 enables the repaired osteochondral tissue to reach the biomechanical level close to native tissue, effectively meeting the functional requirements of joint loading. The HZF4, GHZ/F4, and GH4 groups showed a gradual decrease in these two indices, and the Blank group had the lowest values (Figure 10i,j). This confirms that GHZF4 effectively improves the biomechanical strength of repaired osteochondral tissues, meeting the functional requirements of joint loading.

After micro‐CT and biomechanical testing, rabbit samples were fixed, decalcified, and processed for histological staining (Figure S28). H&E staining demonstrated that the GHZF4 group developed a smooth, continuous cartilage layer with organized chondrocytes at 12 weeks, closely resembling native cartilage; the Blank group showed irregular surfaces, fibrous tissue infiltration, and incomplete defect filling. Safranin O/Fast Green staining verified robust GAG deposition in GHZF4‐repaired cartilage, with a GAG distribution pattern similar to native tissue. In contrast, the Blank and GH4 groups showed weak and discontinuous Safranin O staining, indicating poor cartilage matrix regeneration. These histological results provide direct evidence of enhanced cartilage regeneration in the clinically relevant rabbit model.

CLSM images (Figure 10k) and quantified fluorescence intensity (Figure 10l) showing the expression of CD31 (endothelial cell marker, red fluorescence) and αSMA (pericyte/vascular smooth muscle cell marker, green fluorescence) in repaired osteochondral tissues (DAPI, blue fluorescence, for nuclear labeling) at 12 weeks post‐implantation. The fluorescent intensity of CD31 and αSMA followed the order: GHZF4> HZF4> GHZ/F4> GH4. GHZF4 had the highest fluorescent intensity (abundant neovascularization and vascular maturation), indicating active angiogenesis and early vascular maturation.

## 3 Discussion

Osteochondral repair remains a persistent challenge in regenerative medicine, plagued by the intrinsic trade‐off between mimicking the native tissue's heterogeneous “soft‐to‐hard” gradient, orchestrating spatiotemporally ordered bioactive signaling, and modulating the pathological immune microenvironment. Conventional scaffolds (e.g., layered hydrogels, porous ceramics) often suffer from interfacial delamination, disordered growth factor release, and inadequate mechanical adaptability, failing to recapitulate the endogenous healing cascade and address the oxidative/inflammatory milieu of defects [46]. Although gradient fabrication and controlled release strategies show promise, achieving seamless integration of structural biomimicry, precise signal programming, and multifunctional microenvironment regulation in a single platform remains elusive.

This work presents a biomimetic gradient hydrogel (GHZF4) fabricated via electric field‐driven electrophoretic alignment of BSNF. The nanofiber‐reinforced self‐adaptive hydrogels balance mechanical robustness and dynamic adaptability. Rheological analysis showed >80% G’ recovery after 200% strain due to reversible cross‐linking between SA‐PBA/PVA borate bonds, PVA crystallites, and BSNF entanglement (Figure 2a–j). Rapid stress relaxation (99% within 1000 s) facilitates cell‐mediated matrix remodeling (Figure 3b), consistent with the established principle that substrate stress relaxation regulates stem cell fate [15, 47]. Continuous mechanochemical gradients avoid interfacial delamination, while in situ electrophoretic alignment of BSNF generates gradients in porosity (Figure 2i), mechanical properties (Figure 2n–q), and growth factor (GF) zonation. Although the compressive modulus gradient of GHZF4 (5.35‐47.53 kPa) differs from native osteochondral tissue, its gradient architecture and targeted GF delivery supported near‐native collagen alignment (54.65% parallel surface fibers, 77.69% vertical deep fibers at 10 weeks) (Figure 8f–h), exceeding the performance of many reported gradient scaffolds [12]. The mechanical gradient can be further optimized to better match native tissue by: (1) precisely tuning BSNF content and electric‐field alignment to construct a steeper biomimetic gradient; (2) adjusting cross‐linking density and dynamic interactions to achieve a more natural “soft cartilage‐hard subchondral bone” transition; (3) incorporating bioactive ceramics to enhance the stiffness of the bone‐mimetic region while preserving the softness of the cartilage layer.

Spatiotemporally controlled GF delivery couples angiogenesis, osteogenesis, and chondrogenesis. By encapsulating BMP‐2/TGF‐β3 within ZIF‐8 NPs and localizing them to specific hydrogel layers, GHZF4 achieves programmed release kinetics that recapitulate the endogenous healing sequence. Initial burst release of PDGF‐BB effectively recruits BMSCs (Figure 3) and HUVECs (Figure 4). Sustained ZIF‐8‐mediated delivery of BMP‐2 upregulates osteogenic markers RUNX2 and OPN in the subchondral region, while TGF‐β3 elevates chondrogenic markers SOX9 and ACAN in the cartilage region (Figure 5). Sequential and region‐specific delivery prevents vascular invasion into cartilage, representing an advantage over mixed‐release systems such as GHZ/F4, where premature diffusion of GFs may lead to ectopic calcification (Figure 7a–e). The ZIF‐8 platform extends GF release to 35 days, longer than conventional microsphere systems (14–21 days) [48, 49], supporting enhanced osteogenic capacity (Figure 7 and 8). To clarify the design contributions, GHZF4 integrates both electric‐field‐induced continuous gradient and ZIF‐8‐mediated spatiotemporal GF delivery, whereas HZF4 lacks the continuous gradient structure. Comparison between GHZF4 and HZF4 reveals the contribution of gradient architecture, while comparison between GH4 and HZF4 identifies the role of GFs. Together, these controls verify the synergistic effect of gradient structure and bioactive signal programming. This synergistic strategy offers a feasible approach for engineering complex tissue interfaces.

GHZF4 modulates immune microenvironment to accelerate angiogenesis and osteogenesis. Dysregulated inflammation impairs bone regeneration and contributes to osteochondral repair failure [50]. GHZF4 remodels the pathological microenvironment by inducing macrophage polarization and regulating intercellular crosstalk. Sustained Zn^2+^ release is speculated to potentially modulate the TLR4/NF‐κB pathway, potentially explaining the reduced levels of pro‐inflammatory cytokines (IL‐1β, IL‐6) detected in vitro (Figure 6). This speculation lacks direct protein‐level evidence and remains to be further verified in subsequent studies. ROS‐responsive borate bonds further alleviate oxidative stress and promote M2 polarization. Transcriptomics revealed that GHZF4 potentially downregulates pro‐inflammatory pathways and upregulates signaling related to osteogenesis, angiogenesis, and chondrogenesis. The transcriptomic results only provide potential regulatory pathway clues that are correlated with the observed biological phenotypes, rather than fully validated molecular mechanisms due to the absence of protein‐level verification and pathway inhibition experiments. M2‐polarized macrophages secrete BMP‐2 and VEGF, which amplify stem cell recruitment and ECM deposition [51]. The intrinsic immunomodulatory properties of GHZF4, achieved through controlled Zn^2+^ release and ROS‐responsive borate bonds, establish a novel design principle for immunoregulatory biomaterials. The two animal models were designed with complementary objectives, with inherent differences in species, defect size, and observation period that determine their distinct research values. The rat model was employed to rapidly and systematically validate integrated osteochondral repair efficacy at the tissue and molecular levels, providing comprehensive histological, immunohistochemical, and transcriptomic evidence for early‐to‐mid‐stage cartilage and subchondral bone regeneration. Correspondingly, the rabbit model (adult New Zealand white rabbits; osteochondral defects 4 mm × 4 mm; 12‐ and 24‐week observation) was established to mimic clinical physiological conditions more closely, specifically verifying scaffold‐mediated long‐term repair stability, vascularized subchondral bone regeneration and biomechanical functional recovery under physiological loading. Conclusions derived from each model are model‐specific and complementary, rather than universally applicable.

GHZF4 mimics native osteochondral gradients and signaling pathways, integrating multiple functionalities for cell‐free repair with outcomes superior to cell‐based therapies [52, 53]. Compared with representative continuous gradient hydrogel systems for osteochondral repair [12], the GHZF4 scaffold demonstrates multiple unique advantages: (1) electric‐field‐driven assembly generates seamless compositional, structural, and mechanical gradients that better mimic native osteochondral tissue; (2) ZIF‐8‐mediated spatiotemporal GF delivery achieves sequential coupling of angiogenesis, osteogenesis, and chondrogenesis; (3) integrated self‐healing and tissue‐adhesive properties adapt to the dynamic mechanical environment of joints; (4) the scaffold actively modulates the immune microenvironment by promoting M2 macrophage polarization; (5) it promotes functional osteochondral repair in both rat and rabbit models.

This proof‐of‐concept study has several limitations that warrant further investigation, including the lack of in vivo macrophage phenotyping to verify immunomodulatory effects and direct in vivo quantification of spatiotemporal growth factor release kinetics. Future studies will focus on validating the complete immunomodulation‐angiogenesis‐osteochondrogenesis repair cascade in a single animal model, as well as evaluating long‐term tissue maturation, interface integration, and fatigue resistance under physiological loading conditions. Additional validation studies in large‐animal models will be conducted to further advance the clinical translation of this regenerative strategy. Collectively, this multifunctional gradient system and spatiotemporal delivery strategy represent a promising approach for advancing osteochondral regeneration by engineering biomimetic and physiologically relevant tissue constructs.

## 4 Conclusion

In summary, we developed an electric‐field‐induced continuous gradient hydrogel (GHZF4) integrated with ZIF‐8‐mediated spatiotemporal growth factor delivery for enhanced osteochondral regeneration. The gradient scaffold simultaneously recapitulates the compositional, mechanical, and structural anisotropy of native osteochondral tissue, avoiding interfacial delamination and enabling favorable cell infiltration and matrix remodeling. The sequential and zone‐specific release of growth factors orchestrates angiogenesis, osteogenesis, and chondrogenesis in a spatiotemporally controlled manner, while sustained Zn^2^^+^ release and ROS‐responsive dynamic bonds jointly establish a favorable immune microenvironment by promoting M2 macrophage polarization and suppressing excessive inflammation. The complementary dual animal model system demonstrates repair efficacy in a model‐specific and evidence‐based manner. The rat model provides comprehensive histological and molecular evidence for integrated osteochondral regeneration in a preclinical small animal setting, and the rabbit model validates scaffold‐mediated vascularized subchondral bone reconstruction and biomechanical functional recovery under clinically relevant conditions. By combining structural biomimicry, controlled bioactive factor delivery, and immunomodulation, this scaffold offers a rational design approach for the regeneration of complex, gradient tissues.

## 5 Experimental Section

### 5.1 Materials and Methods

#### 5.1.1 Materials

Silk fibroin was obtained from Meilun Biotechnology Co., Ltd. (Dalian, China). Recombinant rat bone morphogenetic protein‐2 (BMP‐2, Cat#HY‐P7006), recombinant rat transforming growth factor‐β3 (TGF‐β3, expressed in HEK293 cells, Cat#HY‐P7120) and recombinant rat platelet‐derived growth factor‐BB (PDGF‐BB, Cat#HY‐P7278) were purchased from MedChemExpress (USA). Total RNA extraction kits, F12/Dulbecco's modified Eagle's medium (F12/DMEM), penicillin‐streptomycin solution and fetal bovine serum (FBS) were procured from Solarbio (China). The Cell Counting Kit‐8 (CCK‐8) reagent was provided by Dojindo (Kumamoto, Japan). Rabbit polyclonal primary antibodies against Runt‐related transcription factor 2 (RUNX2), osteopontin (OPN), SRY‐box transcription factor 9 (SOX9), aggrecan (ACAN), vascular endothelial growth factor (VEGF), hypoxia‐inducible factor‐1α (HIF‐1α) and glyceraldehyde‐3‐phosphate dehydrogenase (GAPDH) were procured from Abcam (Massachusetts, USA). All antibody dilutions and relevant experimental procedures were performed strictly following the manufacturer's protocols. Alizarin Red S, alkaline phosphatase (ALP) staining solution and ALP assay kit were sourced from Beyotime (China). FITC‐conjugated phalloidin and DAPI were purchased from Sigma‐Aldrich (USA). All remaining chemical reagents were acquired from Macklin (China).

#### 5.1.2 Fabrication of GFs‐Loaded ZIF‐8 NPs

Initially, 22.07 mg of zinc acetate (0.074 mmol, 298.28 g mol^−1^) was dissolved in 2 mL of deionized water. A mixture of 48 mg of MIM (96.13 g mol^−1^, 0.5 mmol), 75 mg of 8arm‐PEG‐NH_2_ (5000, 0.015 mmol), and a given amount of the BMP‐2 was prepared in 2 mL of deionized water while continuously stirring until complete dissolution was achieved. Subsequently, the zinc acetate solution was added to the MIM solution and stirred using ultrasonication for 20 min at room temperature [30]. The ZIF‐8/BMP‐2/8arm‐PEG‐NH_2_ precipitate was collected by centrifugal separation, washed thrice, and dried under vacuum at 37°C. The same procedure was followed to synthesize pure ZIF‐8, ZIF‐8/8arm‐PEG‐NH_2,_ and ZIF‐8/TGF‐β3/8arm‐PEG‐NH_2_. To evaluate the protein loading capacity of the synthesized nanoparticle, 2 mg of the dried NP samples were initially suspended in 2 mL of 1 M hydrochloric acid (diluted with ethanol) for 4 h, facilitating the release of the encapsulated protein via acidic decomposition. The resultant solution was then centrifuged at 10 000 rpm for 10 min, and a Microplate Reader examine the absorption spectra of the supernatant.

$$\begin{array}{ccc} \mathrm{Loading}\;\mathrm{efficiency}\left(\%\right) & = & \left(\mathrm{amount}\;\mathrm{of}\;\mathrm{loaded}\;\mathrm{drug}\right)/\\ & & \left(\mathrm{amount}\;\mathrm{of}\;\mathrm{drug}\;\mathrm{loaded}\;\mathrm{NPs}\right)*100\% \end{array}$$

#### 5.1.3 Fabrication of Hydrogel Precursor

Based on our prior research, Phenylboronic‐acid‐grafted sodium alginate (SA‐PBA) was synthesized by grafting 3‐aminophenyl boronic acid onto the alginate chain using 1‐(3‐dimethylaminopropyl)‐3‐ethyl carbodiimide hydrochloride (EDC**·**HCl) [20]. The beta‐sheet‐rich silk nanofibers (BSNF) were prepared according to our reported protocol. Subsequently, 4 mL of 10% PVA solution, 6 mL of 4.5% SA‐PBA, and 2.5 mL of 2% BSNF solution were mixed and slightly stirred to form the SBPS hydrogel precursor. For the SBPSZ hydrogel precursor, mix 4 mL of 10% PVA solution, 6 mL of 4.5% SA‐PBA, and 2.5 mL of 2% BSNF containing ZIF‐8 NPs.

#### 5.1.4 Fabrication of the Gradient Hydrogel

To fabricate gradient SA‐PBA/PVA/BSNF/ZIF‐8 hydrogels (abbreviated as GHZ), the as‐prepared SBPSZ hydrogel precursor was cast into a custom mold and covered with plastic wrap to prevent moisture evaporation. An electric field was subsequently applied to drive directional migration of BSNF toward the positive anode. The gradient profile of BSNF within the hydrogel matrix can be flexibly modulated by adjusting electric field strength and treatment duration; in this work, 50 V for 30 min was adopted as the optimal condition. Within this parameter range, elevated voltage and prolonged treatment facilitate BSNF migration and ordered alignment, resulting in a steeper gradient, higher mechanical modulus and cross‐linking density, as well as reduced porosity. Finally, the hydrogel was frozen at −30°C for 30 min followed by thawing at room temperature, which promotes secondary cross‐linking of the SBP hydrogel network and stabilizes the BSNF gradient architecture. To study the gradient properties, the hydrogels were evenly divided into seven segments in the direction of the electrical field, named GHZ0, GHZ1, GHZ2, GHZ3, and GHZ4, starting from the cathode to the anode. As the anode and cathode produce bubbles through water electrolysis, the segments located at these two electrodes were excluded from further analysis.

#### 5.1.5 Fabrication of Functional Gradient Hydrogel

To functionalize the gradient hydrogel, BMP‐2‐functionalized ZIF‐8 NPs were incorporated into the SBPS hydrogel precursor loaded with PDGF‐BB to construct a bone layer hydrogel precursor (SBPSP‐ZB). The TGF‐β3‐functionalized ZIF‐8 NPs were incorporated into the SBPS hydrogel precursor to construct the chondral layer hydrogel precursor (SBPS‐ZT). Subsequently, the SBPSP‐ZB hydrogel precursor and SBPS‐ZT hydrogel precursor were poured into opposite sides of the electrolytic cell, separated by a middle divider, and the separator was removed once the power supply was activated. The final hydrogel (GHZF) was obtained according to the preparation method of GHZ hydrogel, with GHZ1, GHZ2, GHZ3, and GHZ4 corresponding to GHZF1, GHZF2, GHZF3, and GHZF4, respectively. Similarly, a hydrogel without bioactive factors and ZIF‐8 was prepared under the same conditions, with the corresponding section labeled as GH4. As a control group, a hydrogel without electrical field treatment was prepared under the same conditions, and the section corresponding to GHZF4 was termed HZF4. Furthermore, a hydrogel containing bioactive factors and ZIF‐8 was prepared under the same conditions, with the corresponding section identified as GHZ/F4.

### 5.2 Characterization of Hydrogels In Vitro

#### 5.2.1 Rheology

The viscosity of the hydrogel precursor solution prior to electric field treatment is a critical determinant of the final gradient distribution. All rheological assays were performed on a rotational rheometer (MCR302, Anton Paar) equipped with a 20 mm cone‐plate geometry at a controlled temperature of 25°C. Amplitude sweeps were first carried out over a strain range of 0.1%–100% to determine the linear viscoelastic region of the hydrogels. Frequency sweep tests were subsequently conducted from 100 to 1 rad s^−1^ within the linear viscoelastic regime, and the storage modulus (G′), loss modulus (G″) and complex viscosity (η) of each sample were collected.

#### 5.2.2 Mechanical Property

The mechanical properties of the hydrogels were evaluated on a universal testing machine (Instron 3344; Instron Co. Ltd., UK) fitted with a 50 N full‐range load cell. In view of the inherent mechanical anisotropy of the oriented hydrogels, all cylindrical specimens (8 mm in diameter, 10 mm in height) were compressed perpendicular to the alignment direction. Tests were conducted at a constant crosshead speed of 2 mm min^−1^ until samples were compressed to above 70% of their original height. The samples, cut into rectangles measuring 40 mm × 10 mm × 8 mm, were subjected to tensile testing parallel to the aligned direction at a rate of 10 mm min^−1^. Five samples were measured for each group.

#### 5.2.3 SEM

Freeze‐dried hydrogel samples were sputtered with a thin gold coating and then imaged using a field‐emission scanning electron microscope (ZEISS Gemini 300, USA) at an accelerating voltage of 20 kV. Surface elemental composition of the lyophilized hydrogels was characterized by X‐ray photoelectron spectroscopy (XPS, Thermo SCIENTIFIC Nexsa, USA).

#### 5.2.4 Porosity

The porosity of the hydrogel was determined using the ethanol weighing method. The lyophilized hydrogel was placed into a test tube containing ethanol for 3 h, and then the porosity of the hydrogel (θ) was determined as follows:

$$\theta =\frac{\omega 1-\omega 3}{\omega 2-\omega 3}\times 100\%$$

where ω1 represents the weight of ethanol and tube, and ω2 and ω3 represent the weight of the whole system with and without the hydrogel, respectively.

#### 5.2.5 Swelling Ratio

The swelling characteristics of hydrogels were determined using the equilibrium swelling index. Briefly, dried hydrogel disks were weighed (W_dry_), and subsequently immersed in PBS at pH 7.4. The disks were shaken on a horizontal shaker at 90 rpm and maintained at 37°C until reaching the swelling equilibrium state. Next, the hydrogel was removed from the solution, the filter paper was used to absorb the free liquid, and the weight was recorded as W_swollen_. The swelling index of hydrogels was quantified using the following equation:

$$\mathrm{Swelling}\;\mathrm{index}=\left(W_{\mathrm{swollen}}-W_{\mathrm{dry}}\right)/W_{\mathrm{dry}}$$

#### 5.2.6 In Vitro Growth Factors Release Profiles of Hydrogels

The release behaviors of BMP‐2, TGF‐β3, and PDGF‐BB from GHZF4 and GHZ/F4 hydrogels were quantified via enzyme‐linked immunosorbent assay (ELISA) kits. Briefly, 0.1 mL of each hydrogel was immersed in 2 mL of PBS (pH 7.4) in 24‐well tissue culture plates, which were incubated on an orbital shaker at 37°C with a constant shaking speed of 90 rpm. Sampling was conducted at predetermined intervals of 1, 3, 5, 7, 14, 21, 28, and 35 days. At each time point, the supernatant was collected, and 1 mL of fresh PBS was replenished into each well. The cumulative release curves were constructed by measuring the protein content in the collected supernatants using ELISA kits strictly following the manufacturer's instructions.

### 5.3 Biocompatibility and Bio‐Function of Hydrogels In Vitro

Bone mesenchymal stem cells (BMSCs) were obtained from the femurs of male Sprague‐Dawley (SD) rats (approximately 40 g) following our established procedures. The cells were passed to the third generation (P3) for further use. Hydrogel samples measuring 8 mm in diameter and 2 mm in height were arranged on glass slide substrates and placed in 48‐well culture plates.

#### 5.3.1 Biocompatibility of Hydrogels In Vitro

Hydrogel extracts were prepared in compliance with the ISO 10993–5 standard. Briefly, hydrogel specimens were immersed in serum‐free DMEM/F12 and incubated with constant orbital shaking at 120 rpm and 37°C for 24 h. The supernatant was harvested and filtered through a 0.22 µm membrane to yield a hydrogel extract at a concentration of 200 mg mL^−1^. Fetal bovine serum was subsequently supplemented to prepare complete extract medium. BMSCs were seeded into 96‐well plates at a density of 1000 cells per well and cultured with extract medium for 1, 3, 5, and 7 days. At each time point, the culture medium was replaced with fresh medium containing 10% CCK‐8 reagent, followed by incubation for 1.5 h at 37°C. Absorbance at 450 nm was detected using a microplate reader (Thermo Fisher Scientific, USA). Cell viability was further visualized via live/dead staining after 3 days of co‐culture.

#### 5.3.2 Cell Adhesion and Cell Recruitment Assay

BMSCs were seeded onto the surface of hydrogel samples at a density of 1 × 10^5^ cells per well and cultured in F12/DMEM for up to 5 days to evaluate cell adhesion behavior. After 5 days of culture, cell‐seeded samples were fixed with 4% paraformaldehyde for 20 min, permeabilized with 0.2% Triton X‐100 for 10 min, and blocked with 2% bovine serum albumin (BSA) for 30 min. Following staining with FITC‐conjugated phalloidin and DAPI, random fields across each sample were imaged under a confocal laser scanning microscope (CLSM; Leica SP8). Live/dead staining was also performed on parallel samples and visualized via CLSM.

For SEM observation, cell‐seeded samples collected at days 5 and 10 were fixed with paraformaldehyde, subjected to gradient ethanol dehydration, and dried via CO_2_ critical point drying. The dried specimens were sputter‐coated with gold and examined under a scanning electron microscope at an accelerating voltage of 20 kV.

#### 5.3.3 Scratch Assay

BMSCs or human umbilical vein endothelial cells (HUVECs) were seeded into 6‐well plates and cultured until 90% confluence. Transwell plate with a 3 µm pore diameter (Corning, #3492, USA) containing 3 specimens in each upper chamber. A uniform scratch was created across each well using a 200 µL pipette tip, followed by washing with PBS to remove detached cells. Cells were then cultured in serum‐free medium, and images of the scratch area were captured at 0 h and 24 h using an inverted optical microscope (Nikon Eclipse TE2000‐U, Kanagawa, Japan). The wound closure rate was calculated as:

$$\mathrm{Migration}\;\mathrm{rate}\left(\%\right)=\left(A_{0}-A_{n}\right)/A_{0}\times 100\%$$

where A_0_ represents the initial scratch area and A_n_ denotes the remaining scratch area at each time point.

#### 5.3.4 Transwell Assay

Cell migration was further evaluated using 24‐well Transwell chambers with 8 µm pore filters (Corning, NY, USA). BMSCs or HUVECs were resuspended in serum‐free medium and seeded into the upper chamber, with one hydrogel specimen placed in each upper chamber. After 24 h of incubation, non‐migrated cells on the upper membrane surface were removed with a cotton swab, while migrated cells on the lower surface were stained with 0.1% crystal violet. Stained cells were imaged and counted to assess migratory capacity.

#### 5.3.5 Tube Formation Assay

HUVECs were resuspended in complete hydrogel extract medium and seeded onto Matrigel‐coated 24‐well plates at a density of 3 × 10^4^ cells mL^−1^. After 6 h of incubation at 37°C, capillary‐like tube formation was observed and imaged under an inverted microscope. The number of junctions and total tube length were quantified using ImageJ software.

#### 5.3.6 Chondrogenic‐Osteogenic Differentiation

Passage 3 (P3) BMSCs were seeded into 24‐well plates at a density of 1 × 10^4^ cells per well. After cell adhesion, the culture medium was replaced with chondrogenic‐osteogenic co‐induction extract medium, which consisted of F12/DMEM supplemented with 10% FBS, 10 ng mL^−1^ recombinant human TGF‐β3, 100 U mL^−1^ penicillin‐streptomycin (P/S), 100 nmol L^−1^ dexamethasone, 91.5 µg mL^−1^ ascorbic acid 2‐phosphate, 10 mmol L^−1^ β‐sodium glycerophosphate, and 40 ug/mL L‐proline. The medium was refreshed every 3 days.

Osteogenic differentiation was evaluated via alkaline phosphatase (ALP) and Alizarin Red S (ARS) staining. After 14 and 28 days of co‐culture, cells were fixed with 10% formalin and stained with ALP staining solution or ARS solution for 30 min, followed by microscopic observation. Staining intensity was quantified using ImageJ software.

Chondrogenic differentiation was assessed via Alcian blue staining. After 14 days of induction, BMSCs were rinsed with PBS, fixed with 4% paraformaldehyde, and stained with Alcian blue solution for 30 min at room temperature. Stained samples were observed and imaged under an optical microscope.

#### 5.3.7 Immunofluorescence Double‐Staining

P3 BMSCs (4 × 10^5^ cells/well) were seeded onto the hydrogel surface in 48‐well plates and cultured for 24 h in F12/DMEM containing 10% FBS and 100 units mL^−1^ P/S. Cells were then maintained in chondrogenic‐osteogenic co‐induction medium for 28 days, with medium renewal every 3 days. Dual immunofluorescence staining was performed to identify chondrogenic and osteogenic differentiation, targeting Runt‐related transcription factor 2 (Runx2)/SRY‐box transcription factor 9 (SOX9) and osteopontin (OPN)/aggrecan (ACAN), respectively. Briefly, samples were rinsed with PBS, fixed with 4% paraformaldehyde for 15 min, and permeabilized with 0.5% (v/v) Triton X‐100/PBS for 5 min. After three washes with PBS, samples were blocked with 5% (w/v) BSA blocking buffer for 1 h, then incubated with primary antibodies diluted in 1% BSA/PBS overnight at 4°C. Following three 5‐min washes with PBS, samples were incubated with corresponding secondary antibodies diluted in 1% BSA/PBS for 40 min at room temperature, protected from light. Nuclei were counterstained with DAPI for 5 min, followed by three final PBS washes. Fluorescent images were captured via CLSM and analyzed using ImageJ.

#### 5.3.8 Evaluation of the Repolarization of Macrophages

RAW 264.7 murine macrophage cells (Catalog No. CL‐0190) were purchased from Procell Life Science & Technology Co., Ltd. Cells were seeded into 6‐well plates and cultured in RAW 264.7‐specific complete medium (Procell) until 80% confluence, then stimulated with lipopolysaccharide (LPS; 1 µg mL^−1^) after adhesion. The experimental groups were set as follows: untreated RAW 264.7 cells under basal culture (M2 control group), cells treated with 1 µg mL^−1^ LPS alone (LPS group), LPS‐stimulated cells co‐cultured with GH4 hydrogel (LPS + GH4 group), and LPS‐stimulated cells co‐cultured with GHZF4 hydrogel (LPS + GHZF4 group). After 24 h of co‐culture, the expression of M2 polarization‐related genes was detected via quantitative real‐time polymerase chain reaction (RT‐qPCR; primer sequences listed in Table S1). M2‐associated surface proteins were further analyzed via flow cytometry and western blotting.

#### 5.3.9 Antioxidant Study

Intracellular reactive oxygen species (ROS) levels were measured to evaluate the antioxidant capacity of the hydrogels. RAW 264.7 cells were seeded into 24‐well plates and pretreated with hydrogel extracts from each group for 24 h, followed by stimulation with 1 µg mL^−1^ LPS for 4 h. Cells were then incubated with DCFH‐DA probe (Beyotime, China) and Hoechst (Beyotime, China) at 37°C for 30 min. Fluorescent images were captured using an inverted fluorescence microscope. Cells cultured in normal growth medium served as the negative control (NTC), while cells treated with 1 µg mL^−1^ LPS alone served as the positive control (PTC).

#### 5.3.10 Osteogenic Activity Evaluation in Osteoblasts

MC3T3‐E1 cells were seeded into 6‐well plates and allowed to adhere overnight. Cells were then treated with four different media: fresh complete DMEM, LPS‐containing medium, LPS + GH4 conditioned medium (supernatant from LPS + GH4‐treated macrophages diluted with fresh complete DMEM at a 1:2 ratio), and LPS + GHZF4 conditioned medium (supernatant from LPS + GHZF4‐treated macrophages diluted with fresh complete DMEM at a 1:2 ratio). The culture medium was refreshed every 2–3 days. After 7 days of culture, ALP activity was detected using an ALP assay kit following the manufacturer's instructions, and cells were observed under an inverted microscope. After 21 days, ARS staining was performed to visualize osteogenic mineralized nodules.

#### 5.3.11 RT‐qPCR Analysis

After 14 days of co‐culture with hydrogels, total RNA was extracted from BMSCs and HUVECs using Trizol reagent, and reverse transcription was performed using a RevertAid first‐strand cDNA synthesis kit (Takara, Japan) according to the manufacturer's protocol. Quantitative real‐time PCR (RT‐qPCR) was conducted on an ABI StepOne Plus real‐time PCR system (Applied Biosystems, USA) using a SYBR Green PCR kit (Takara, Japan) to determine the expression levels of target genes. Primer sequences are provided in Table S1. All samples were analyzed in triplicate, and relative gene expression was calculated using the 2^−^ΔΔCt method, with GAPDH serving as the internal reference gene.

#### 5.3.12 Western Blot Assay

The expression levels of angiogenic, chondrogenic, and osteogenic proteins were detected via western blotting. After 14 days of chondrogenic‐osteogenic co‐culture with hydrogels, BMSCs and HUVECs were lysed, and total protein concentration was determined using a BCA protein assay kit. Equal amounts of protein (30 µg per lane) were separated via SDS‐PAGE and transferred onto polyvinylidene difluoride (PVDF) membranes on ice. Membranes were blocked with 5% skim milk in Tris‐buffered saline with Tween 20 (TBST) for 1 h to block non‐specific binding, then washed three times with TBST and incubated with primary antibodies overnight at 4°C. After washing, membranes were incubated with corresponding horseradish peroxidase‐conjugated secondary antibodies (1:10,000 dilution) for 1 h at room temperature. Target protein bands were visualized using enhanced chemiluminescence (ECL) reagent. Band optical density was analyzed using Bio‐Rad image analysis software and further quantified via ImageJ. Glyceraldehyde‐3‐phosphate dehydrogenase (GAPDH) was used as the internal loading control.

### 5.4 Biocompatibility and Bio‐Function of Hydrogels In Vivo

All animal experimental protocols were approved by the Laboratory Animal Ethics Committee of the Ninth People's Hospital, Shanghai Jiao Tong University School of Medicine (Approval No. SH9H‐2023‐A863‐1), and all procedures were performed in strict compliance with ethical guidelines for laboratory animal care and use.

#### 5.4.1 Rat Model and Surgical Procedure

8‐week‐old male Sprague‐Dawley (SD) rats were randomly assigned to five groups: Blank, HZF4, GH4, GHZ/F4, and GHZF4. After general anesthesia, the skin over the left knee was sterilized with iodophor. A 1 cm longitudinal incision was made at the medial parapatellar region, and the medial collateral ligament was dissected to expose the medial meniscus. A cylindrical osteochondral defect (1.6 mm in diameter, 3 mm in depth) was created at the trochlear groove of the distal femur using a dental micromotor. Hydrogel specimens were implanted into the defects in the four treatment groups, while the Blank group received no implant. All gradient hydrogels were oriented with the softer layer facing the cartilage side and the stiffer layer aligned with the subchondral bone, to recapitulate the hierarchical architecture of native osteochondral tissue. Rats were euthanized at 5 and 10 weeks post‐implantation for gross observation, micro‐CT scanning, and International Cartilage Repair Society (ICRS) macroscopic scoring. In addition, major organs (heart, lung, liver, kidney, and spleen) were harvested at 2 weeks post‐surgery for H&E staining to verify the in vivo biosafety of the composite hydrogels.

#### 5.4.2 Rabbit Model and Surgical Procedure

Adult male New Zealand white rabbits weighing 3.0–3.5 kg were used for the in vivo validation study. Rabbits were randomly divided into six groups, with both knee joints utilized for defect construction to minimize animal usage: Blank, GH4, GHZ/F4, HZF4, GHZF4, and Natural group. After anesthesia, the knee joint was exposed via parapatellar incision and patellar dislocation. A cylindrical osteochondral defect (4 mm in diameter, 4 mm in depth) was prepared at the femoral trochlear groove using a trephine bur. Size‐matched hydrogel specimens were then implanted into the defect site. Passive flexion‐extension of the operated knee was performed to confirm stable fixation of the implanted scaffolds. The incision was closed layer by layer with 4‐0 sutures, and prophylactic antibiotics were administered intramuscularly postoperatively. All rabbits were housed individually with free access to standard chow and water, and were euthanized at 12 and 24 weeks for subsequent analyses.

#### 5.4.3 Micro‐CT Analysis

All harvested joint specimens were scanned using a micro‐CT system (Hiscan UM, China) with an isotropic resolution of 11 µm. Three‐dimensional reconstructed images were generated via CT‐Volume software, and multi‐planar visualization (coronal, sagittal, and trans‐axial) of the defect site and adjacent native tissue was performed with Data Viewer software. A circular region of interest (ROI) matching the original defect dimensions was defined for quantitative analysis: 1.6 mm diameter × 3 mm depth for rat samples, and 4 mm diameter × 4 mm depth for rabbit samples. Key bone morphometric parameters including bone mineral density (BMD), bone volume fraction (BV/TV), trabecular thickness (Tb.Th), and trabecular number (Tb.N) were quantified. All measurements and analyses were performed independently by three blinded investigators.

#### 5.4.4 Biomechanical Properties of Neo‐Osteochondral Tissue

Nanoindentation tests were conducted to determine the mechanical properties of regenerated osteochondral tissue at 10 weeks post‐implantation for rats and 24 weeks for rabbits. Measurements were primarily performed on the surface of neo‐cartilage within the defect region. Tests were carried out on a Tribo‐Indenter system (Hysitron TI 950, USA) equipped with a conospherical diamond probe with a 400 nm radius of curvature. Five test points (anterior, posterior, medial, lateral, and central) were selected for each sample. A trapezoidal load profile was applied at each site, consisting of a 10 s loading phase, 2 s hold phase, and 10 s unloading phase. A force‐controlled mode was adopted with a maximum indentation depth of 500 nm. Reduced modulus and hardness were calculated via linear fitting of the load‐displacement curves. In addition, surface topography of the repaired tissue was acquired via scanning probe microscopy (SPM) integrated with the nanoindentation system.

#### 5.4.5 Histology and Immunohistochemistry Staining and Histomorphometric Scoring

Harvested femoral specimens were fixed in 10% neutral buffered formalin for at least 5 days, followed by decalcification in EDTA solution for 30 days. After gradient ethanol dehydration and paraffin infiltration, specimens were embedded and sectioned longitudinally at 5 µm thickness. Sections were stained with hematoxylin and eosin (H&E) for general tissue morphology, and Safranin O/Fast Green for glycosaminoglycan distribution. The defect region (including cartilage and subchondral bone layers) and surrounding native tissue were observed under a light microscope (Nikon Eclipse Ci‐L, Japan). Histological repair quality was evaluated using the Wakitani semi‐quantitative scoring system [54], with a maximum score of 14 points representing the poorest repair outcome (detailed in Table S2). Four blinded independent evaluators scored representative sections based on both the Wakitani histological criteria and the ICRS macroscopic scoring system (Table S3). Picrosirius Red staining was performed to visualize collagen fibers, and the orientation distribution of collagen fibers in the superficial and deep zones of regenerated articular cartilage was analyzed using the Directionality plugin in ImageJ. For rabbit osteochondral specimens, immunofluorescence staining for CD31 and α‐SMA was performed following standard protocols to assess vascular maturation at 12 weeks post‐implantation.

#### 5.4.6 RNA‐Seq and Bioinformatics Analysis

To explore the underlying molecular mechanisms of osteochondral repair, total RNA was extracted from peri‐defect tissues using TRIzol reagent according to the manufacturer's instructions. After RNA purification, reverse transcription and library construction, high‐throughput RNA sequencing (RNA‐seq) was performed. All downstream data analyses, including Gene Ontology (GO) functional enrichment and Kyoto Encyclopedia of Genes and Genomes (KEGG) pathway enrichment analysis, were conducted on the NovoMagic Cloud Platform.

### 5.5 Statistical Analysis

Statistical analyses were conducted using SPSS 16.0 software (USA). All data are presented as mean ± standard deviation (SD). One‐way analysis of variance (one‐way ANOVA) was employed for multi‐group comparisons, as all experiments were designed as single‐factor comparisons conducted at matched time points under identical experimental conditions. All assays were performed with three independent biological replicates (*N* = 3). *P* ≤ 0.05 was considered statistically significant unless otherwise specified. Statistical significance levels are marked as *p < 0.05, **p < 0.01 and ***p < 0.001.

## Author Contributions

X.L.N.: writing – original draft, writing – review & editing, methodology, formal analysis, investigation. S.Z.X.: funding acquisition, supervision, resources, software, visualization. D.H.: methodology, investigation, writing – review & editing. X.S.W.: visualization, validation. Y.W.C.: formal analysis, visualization. X.D.S.: data curation, software. N.D.: writing – review & editing, conceptualization. X.M.L.: conceptualization, supervision, project administration, funding acquisition, resources.

## Conflicts of Interest

The authors declare no conflicts of interest.

## Supporting information




**Supporting file**: advs76469‐sup‐0001‐SuppMat.docx.

## Data Availability

The data that support the findings of this study are available on request from the corresponding author. The data are not publicly available due to privacy or ethical restrictions.
