# Supplementary material for: Biomimetic Gradient Hydrogels Regulate Osteochondral Regeneration Microenvironment Remodeling via Spatiotemporal Programming Engineering
Source: Adv Sci (Weinh). 2026 Jul 8:e76469. Online ahead of print. doi: 10.1002/advs.76469 (PMC13345314; doi:10.1002/advs.76469)
Supplement: Supplementary file 1 — Supporting file: advs76469‐sup‐0001‐SuppMat.docx. [file ADVS-9999-e76469-s001.docx]

Biomimetic Gradient Hydrogels Regulate Osteochondral Regeneration Microenvironment Remodeling via Spatiotemporal Programming Engineering

Xiaolian Niu^1,3^, Shengzhao Xiao^2^*, Di Huang^3^, Xuesong Wang^4^, Yanwei Cao^4^, Xiaodan Sun^5^, Nicholas Dunne^6^, Xiaoming Li^1^*

^1^Key Laboratory for Biomechanics and Mechanobiology of Ministry of Education, Key Laboratory of Innovation and Transformation of Advanced Medical Devices of Ministry of Industry and Information Technology, National Medical Innovation Platform for Industry-Education Integration in Advanced Medical Devices (Interdiscipline of Medicine and Engineering), Beijing Advanced Innovation Center for Biomedical Engineering, School of Biological Science and Medical Engineering, Beihang University, Beijing 100083, China

^2^Department of Orthodontics, Shanghai Ninth People’s Hospital, Shanghai Jiao Tong University School of Medicine; College of Stomatology, Shanghai Jiao Tong University; National Center for Stomatology; National Clinical Research Center for Oral Diseases; Shanghai Key Laboratory of Stomatology; Shanghai Research Institute of Stomatology, Shanghai 200011, China

^3^Department of Biomedical Engineering, Research Center for Nano-biomaterials & Regenerative Medicine, College of Artificial Intelligence, Taiyuan University of Technology, Taiyuan 030024, China

^4^Sports Medicine Service, Beijing Jishuitan Hospital, Capital Medical University, Beijing 100035, China.

^5^Key Laboratory of Advanced Materials of Ministry of Education, Tsinghua University, Beijing 100084, China

^6^Centre for Medical Engineering Research, School of Mechanical and Manufacturing Engineering, Dublin City University, Stokes Building, Collins Avenue, Dublin 9, Ireland

*Corresponding authors: Xiaoming Li, E-mail: x.m.li@hotmail.com; Shengzhao Xiao, E-mail: [shengzhaoxiao@shsmu.edu.cn](mailto:shengzhaoxiao@shsmu.edu.cn)

***Supplementary information***

***
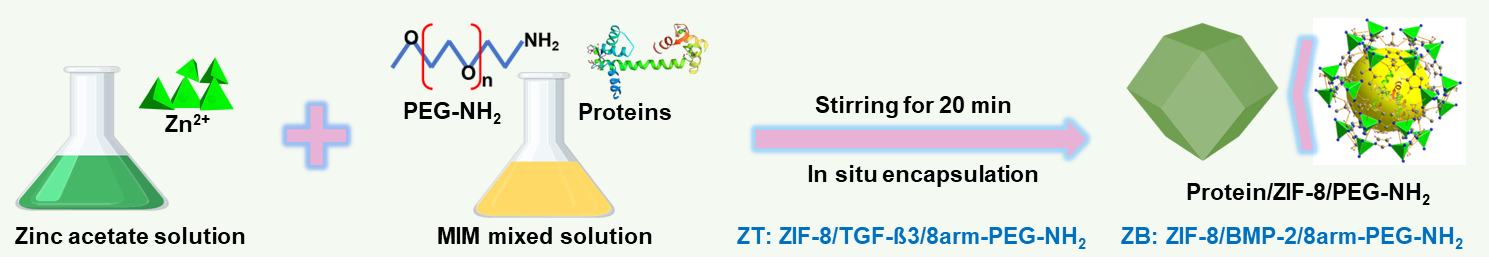
***

**Figure S1.** **The fabrication process of GFs-loaded ZIF-8.**

**
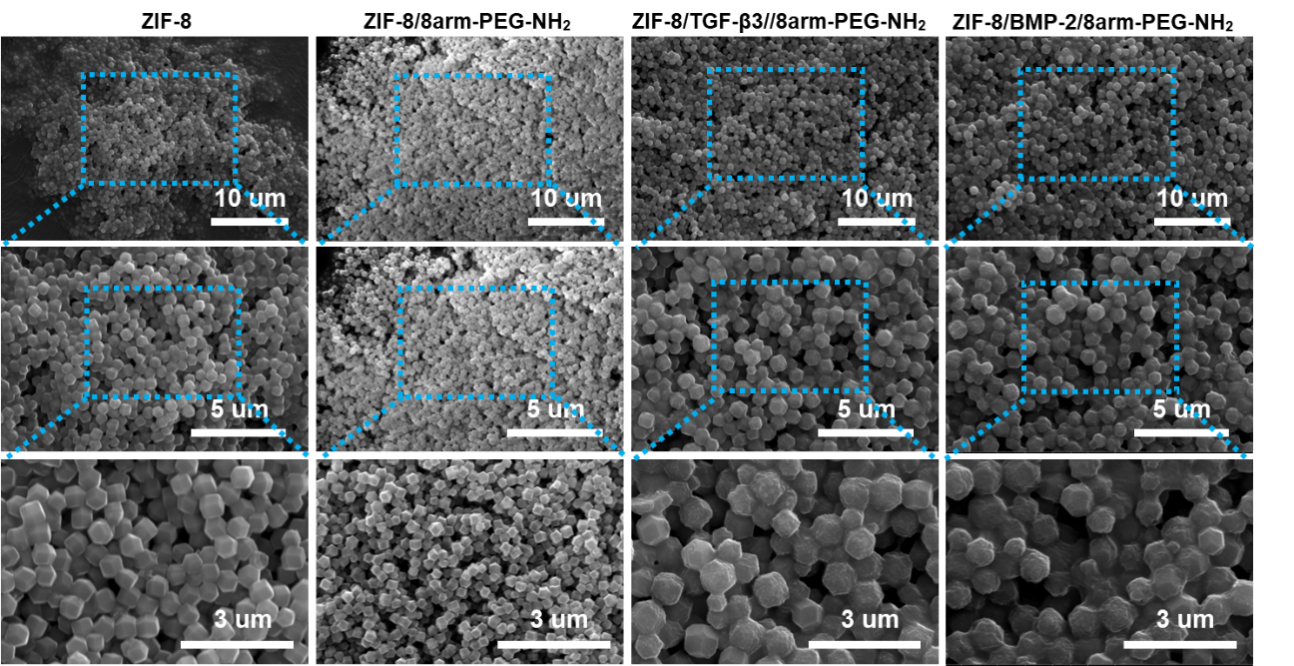
**

**Figure S2.** **SEM images of nanoparticles.**


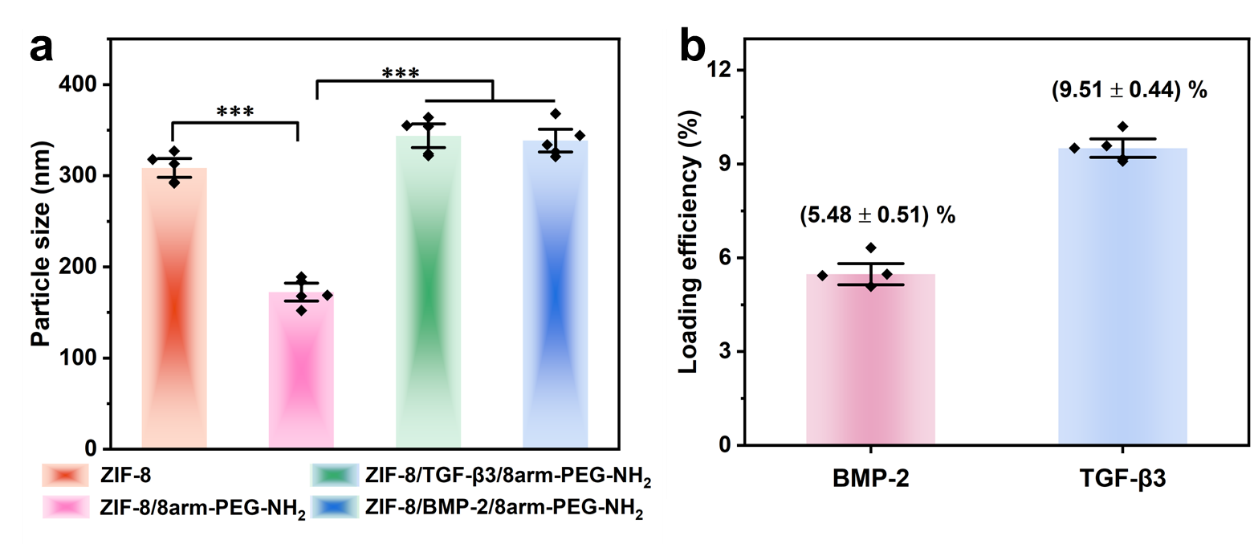


**Figure S3.** **Characterization of** **GFs-loaded ZIF-8 NPs. a)** ImageJ analyzed particle size based on SEM images; **b)** The loading efficiency of NPs.


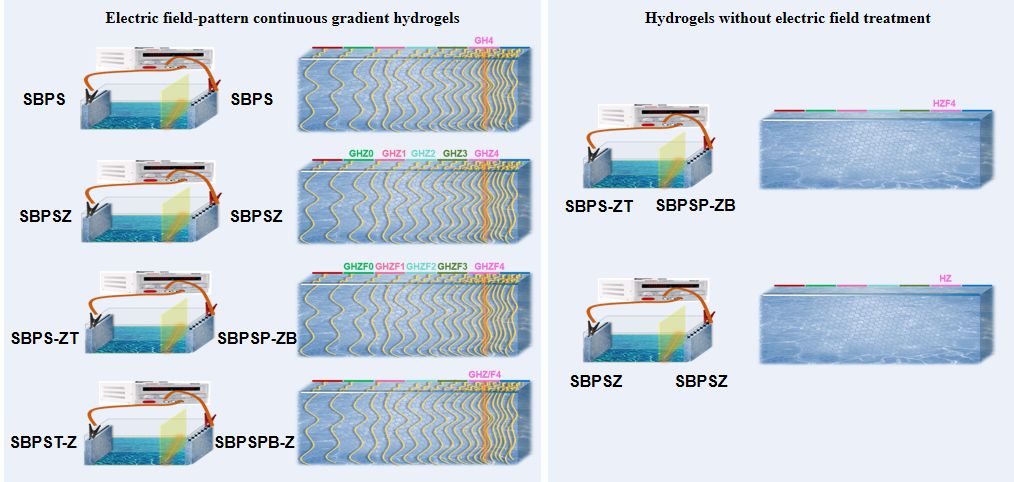


**Figure S4.** **The hydrogels were cut into seven sections along the electrical field direction to study the gradient properties.**


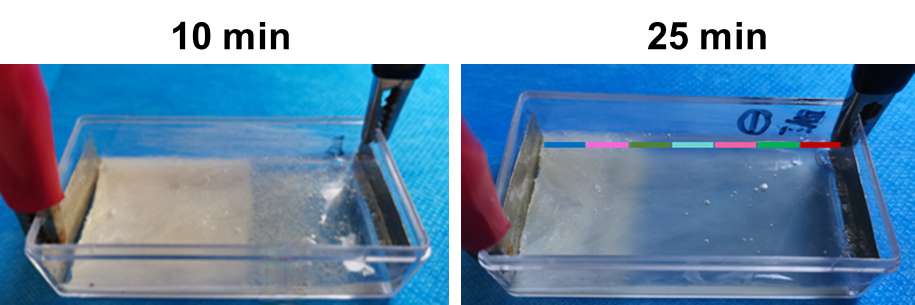


**Figure S5.** **Photograph of gradient hydrogels under an electric field over time.**

**Figure S6.** **The composition of hydrogels for experiments.**


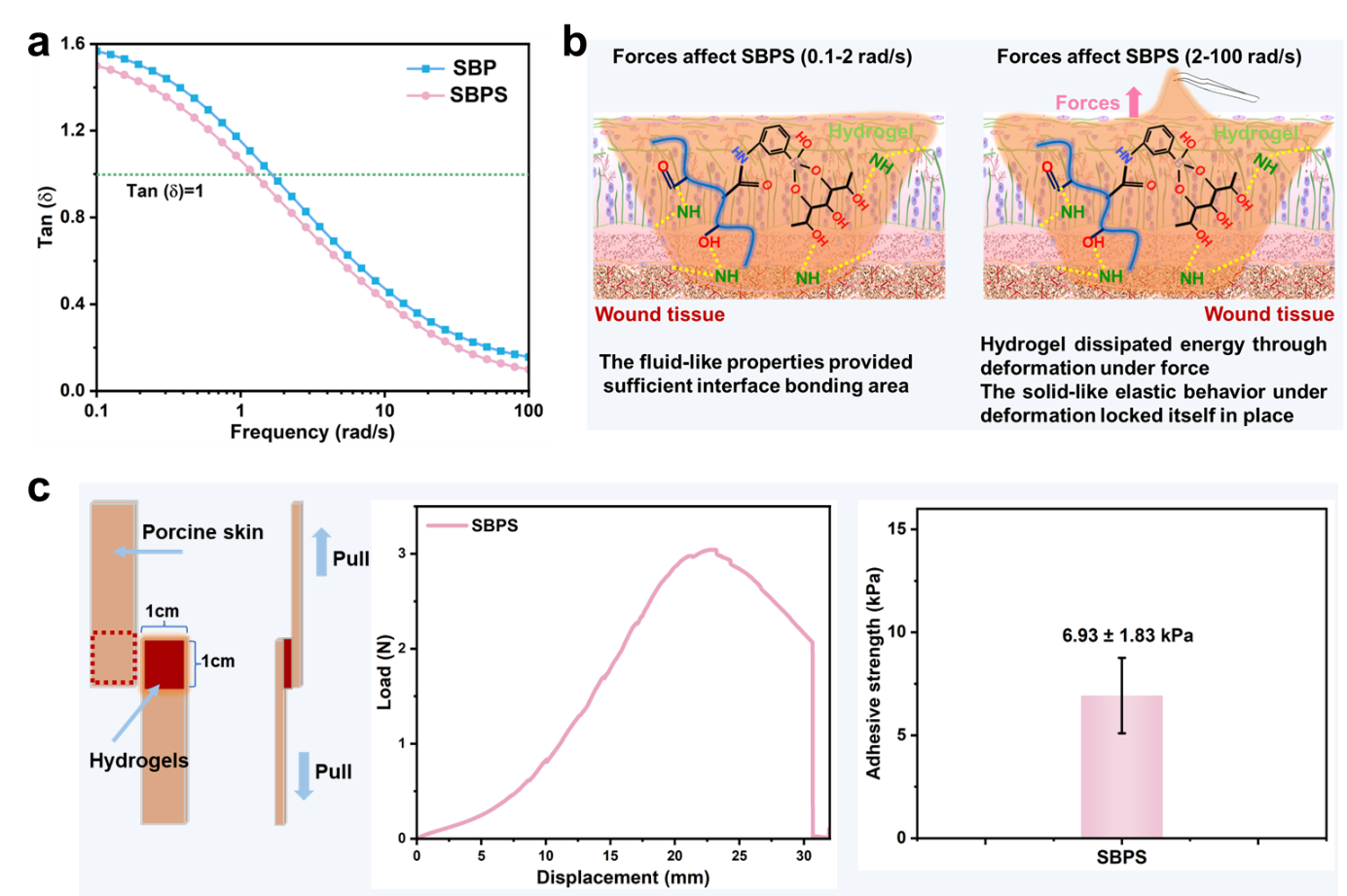


**Figure S7.** **Physical characterization of the SBPS hydrogels.** a) Tan (𝛿) showed the frequency-dependent viscoelasticity required for hydrogel adhesion; b) The adhesion mechanism of the hydrogels; c) The lap shear test for the hydrogel adhesion to porcine skin.


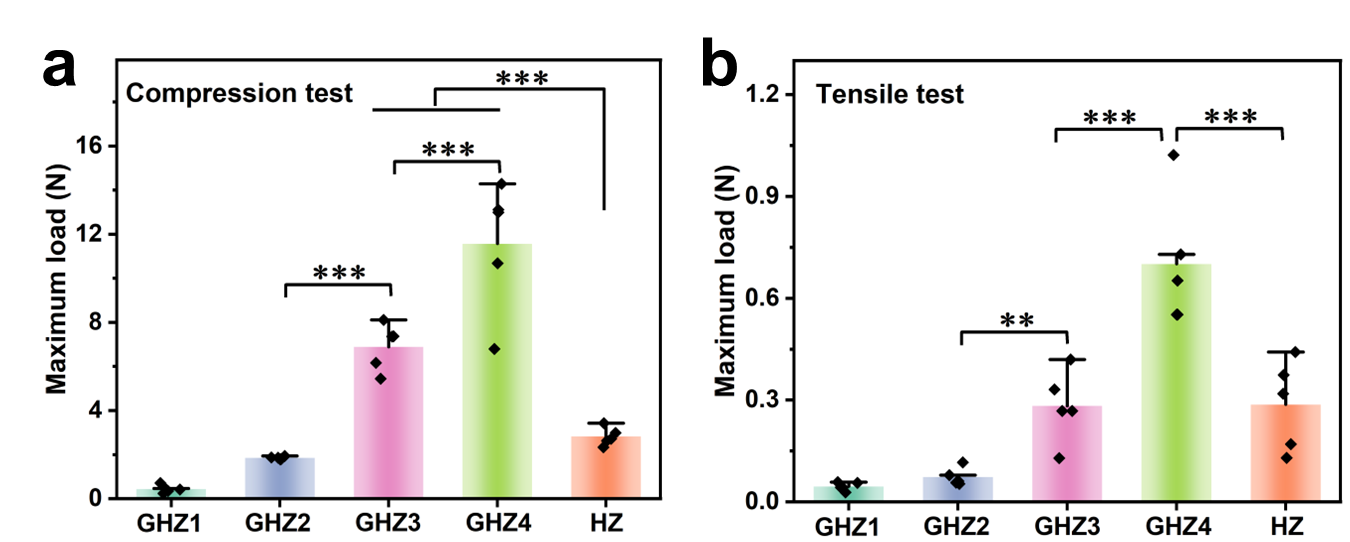


**Figure S8.** **The maximum load based on the strain-stress curve of the compressed test (a) and tensile test (b).**


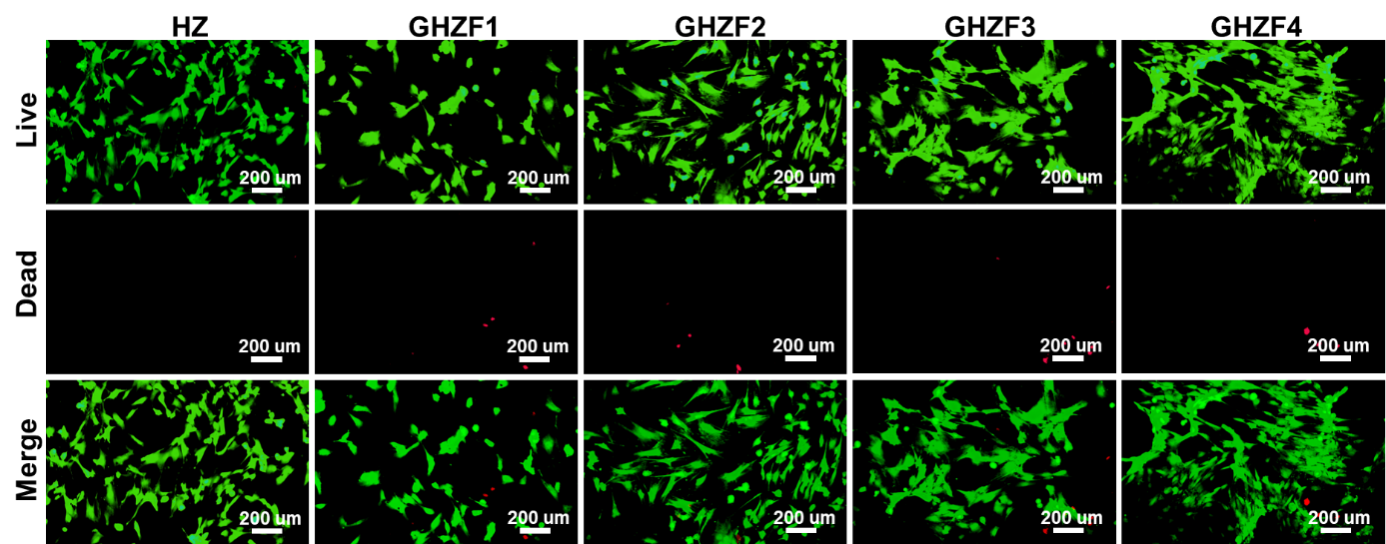


**Figure S9.** ***In vitro* biocompatibility of the hydrogel system.** Representative fluorescence images of live-dead staining for BMSCs after 3 days.


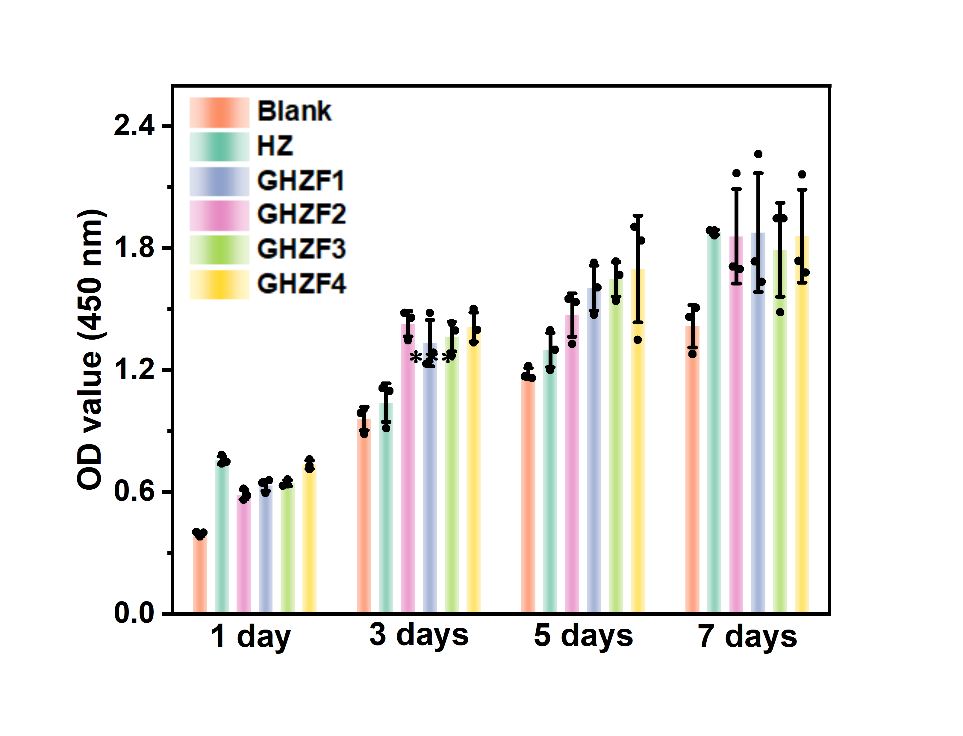


**Figure S10.** **Cell proliferation on hydrogels.**


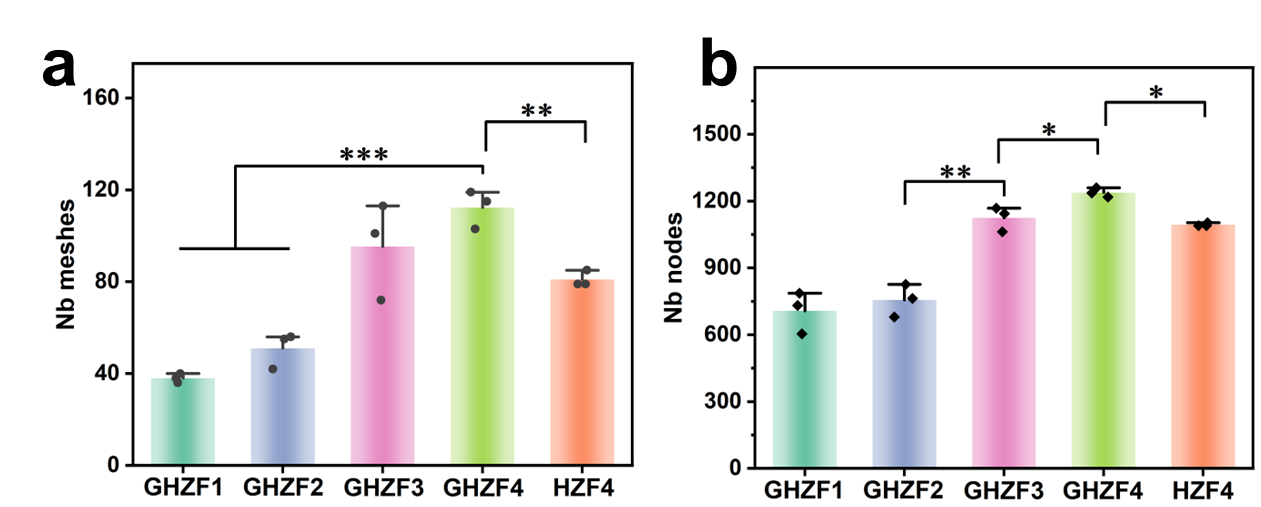


**Figure S11.** **Quantitative analysis of the tube formation ability using ImageJ software.**


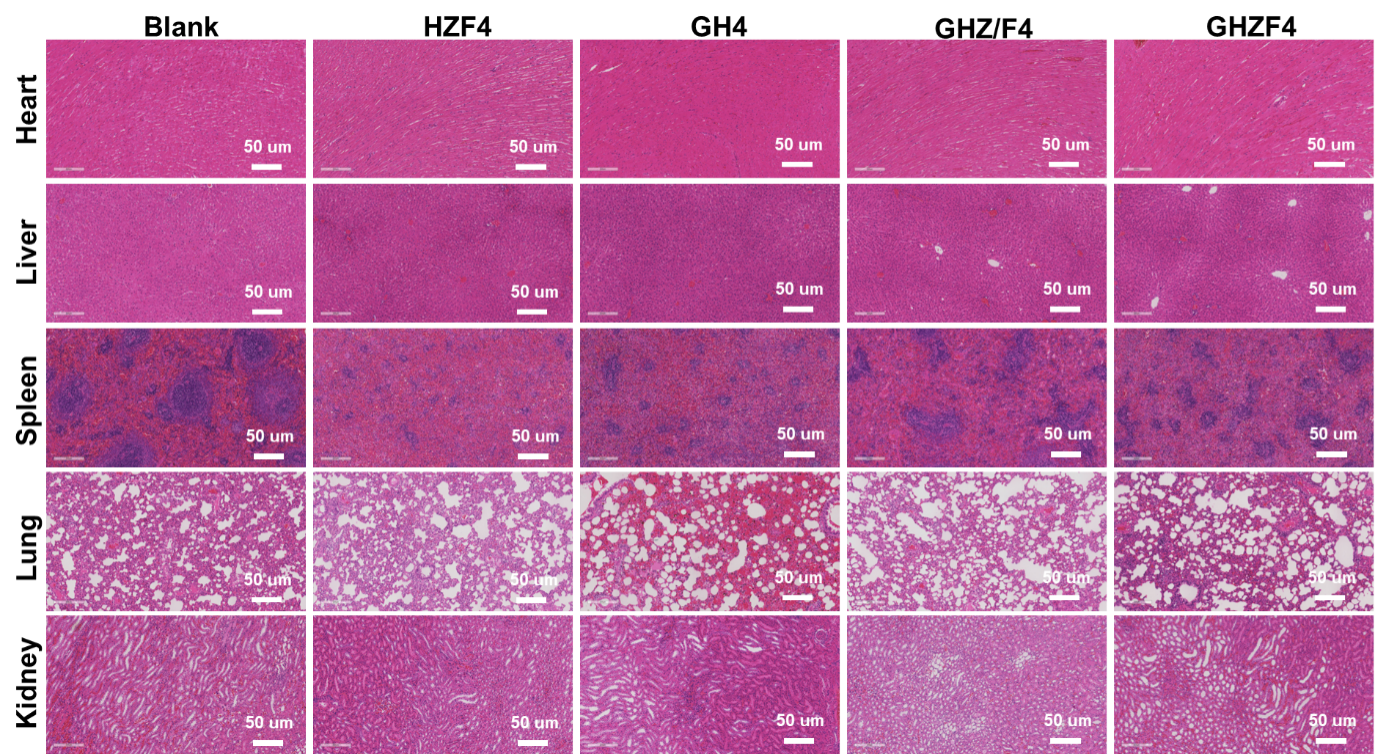


**Figure S12.** ***In vivo* biocompatibility of the hydrogel system.** Representative H&E images of heart, liver, spleen, lung, and kidney tissues at 2 weeks after hydrogel implantation.


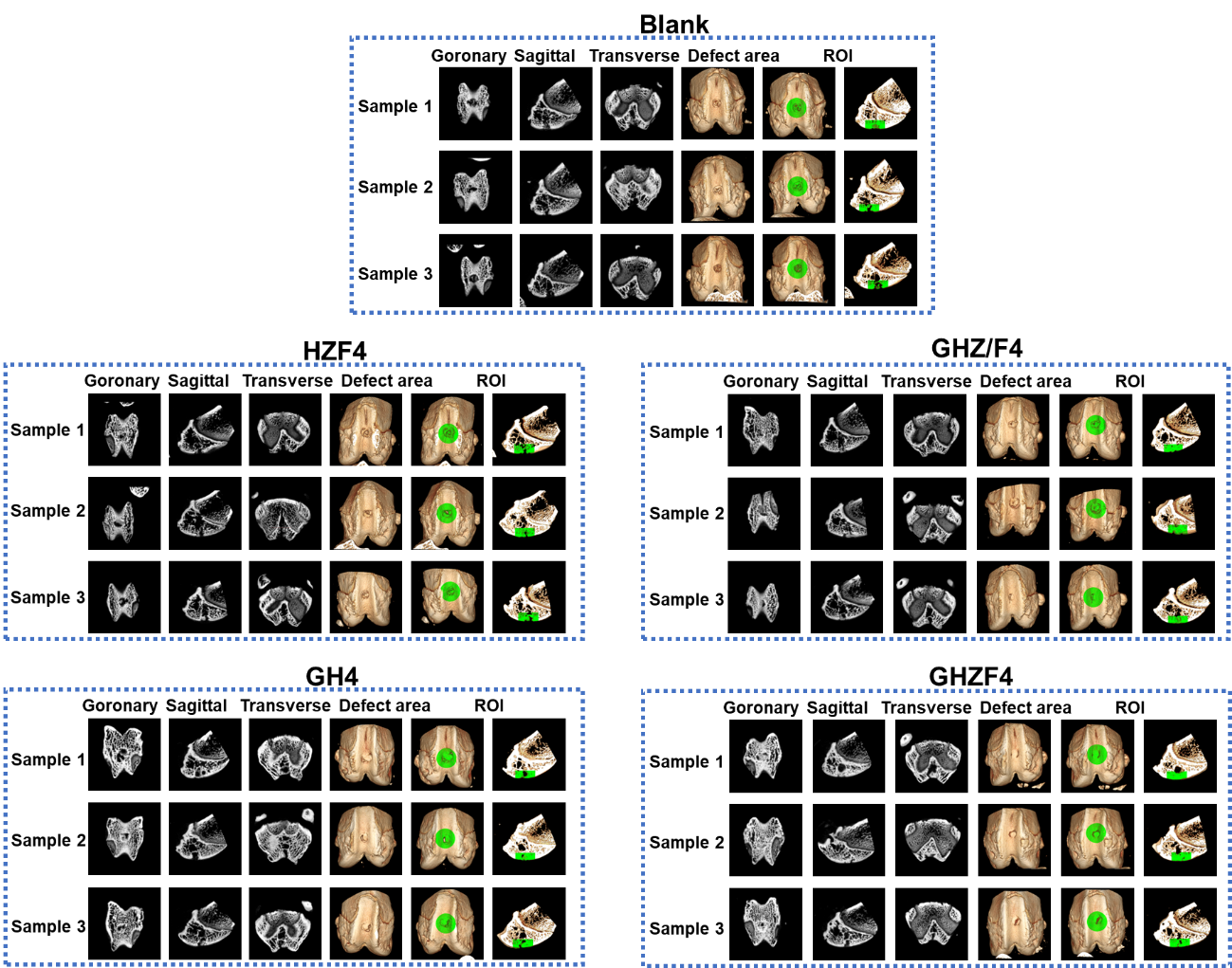


**Figure S13.** **The horizontal, coronal, and 3D reconstructed Micro-computed tomography (Micro-CT) scanning images of osteochondral defects and regenerated osteochondral tissue induced by hydrogels after 5 weeks.**


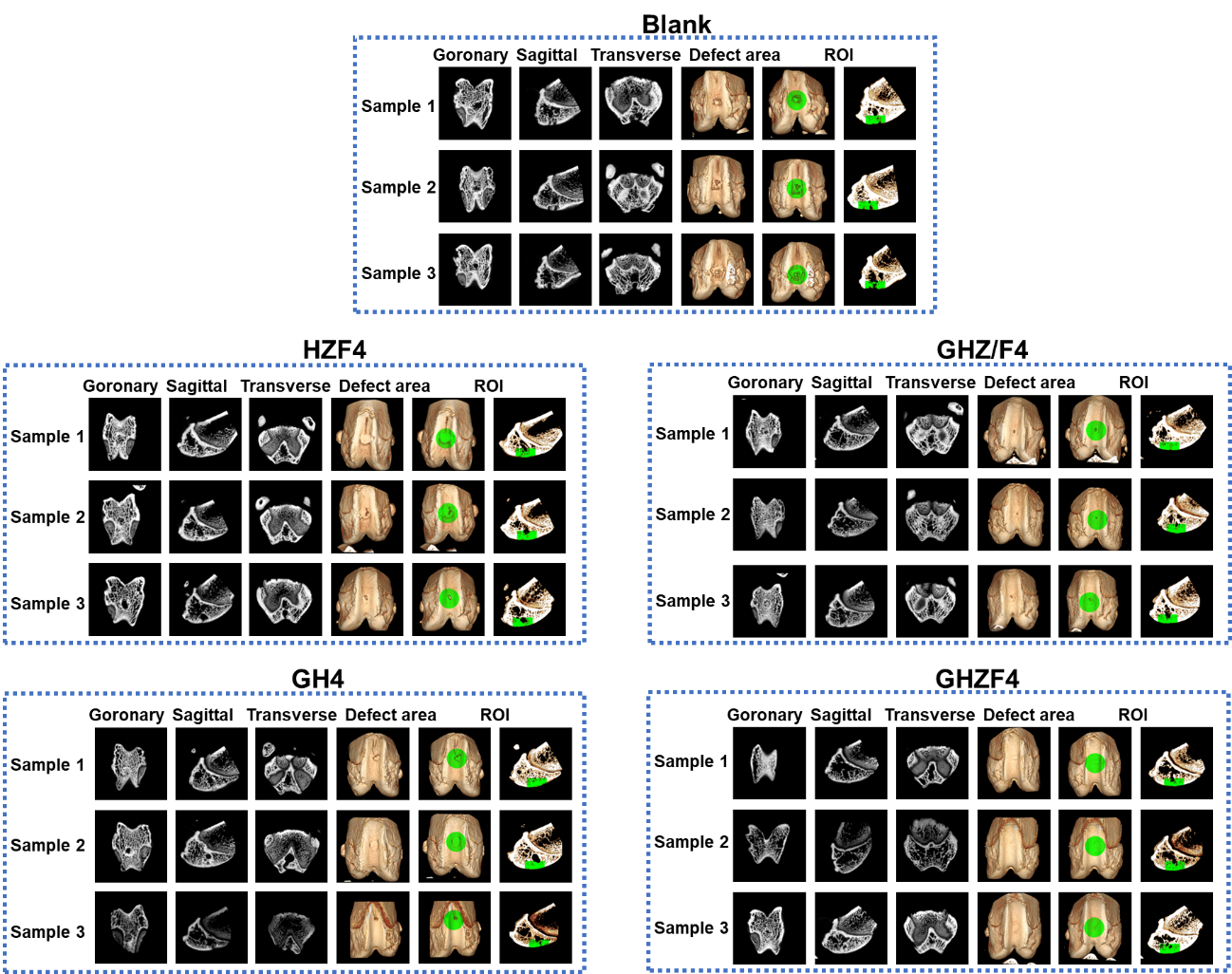


**Figure S14.** **The horizontal, coronal, and 3D reconstructed Micro-computed tomography (Micro-CT) scanning images of osteochondral defects and regenerated osteochondral tissue induced by hydrogels after 10 weeks.**


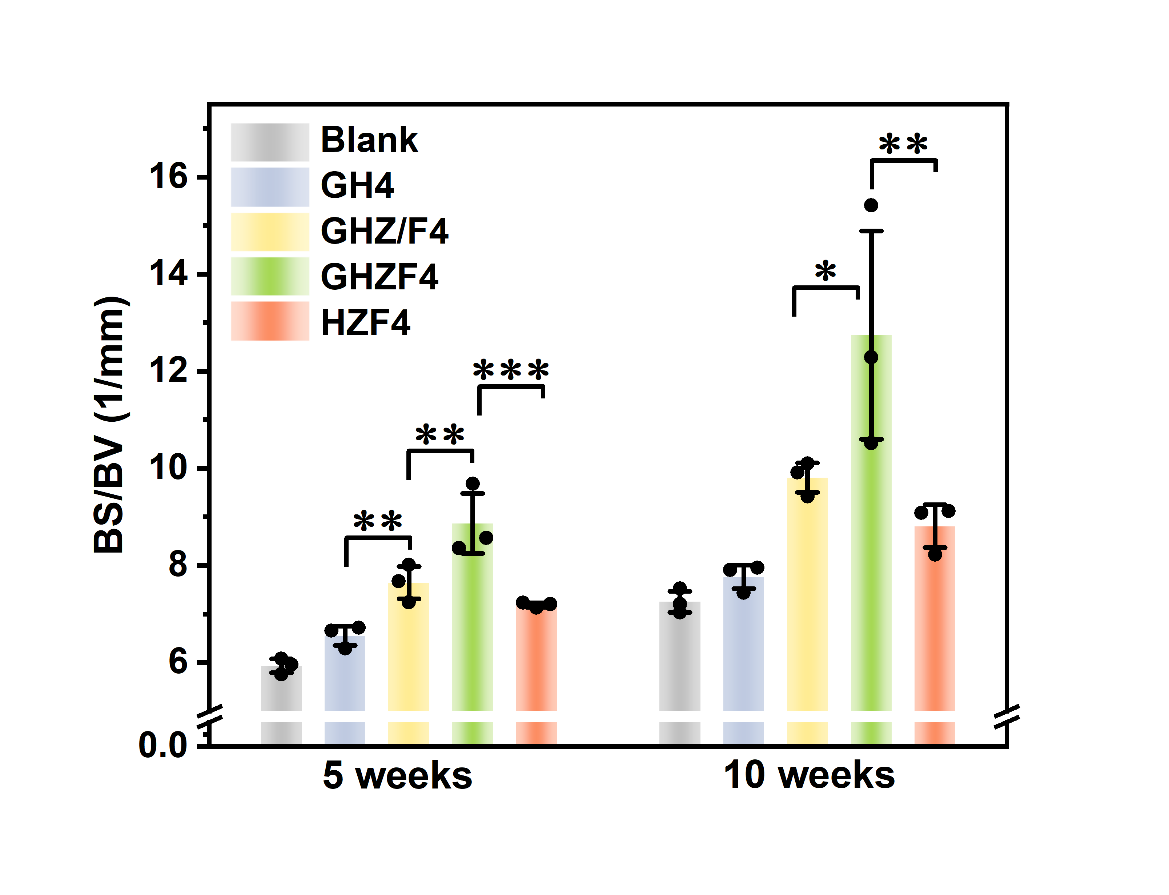


**Figure S15.** **Bone mass analysis of the obtained samples with quantitative micro-CT for BS/BV.**


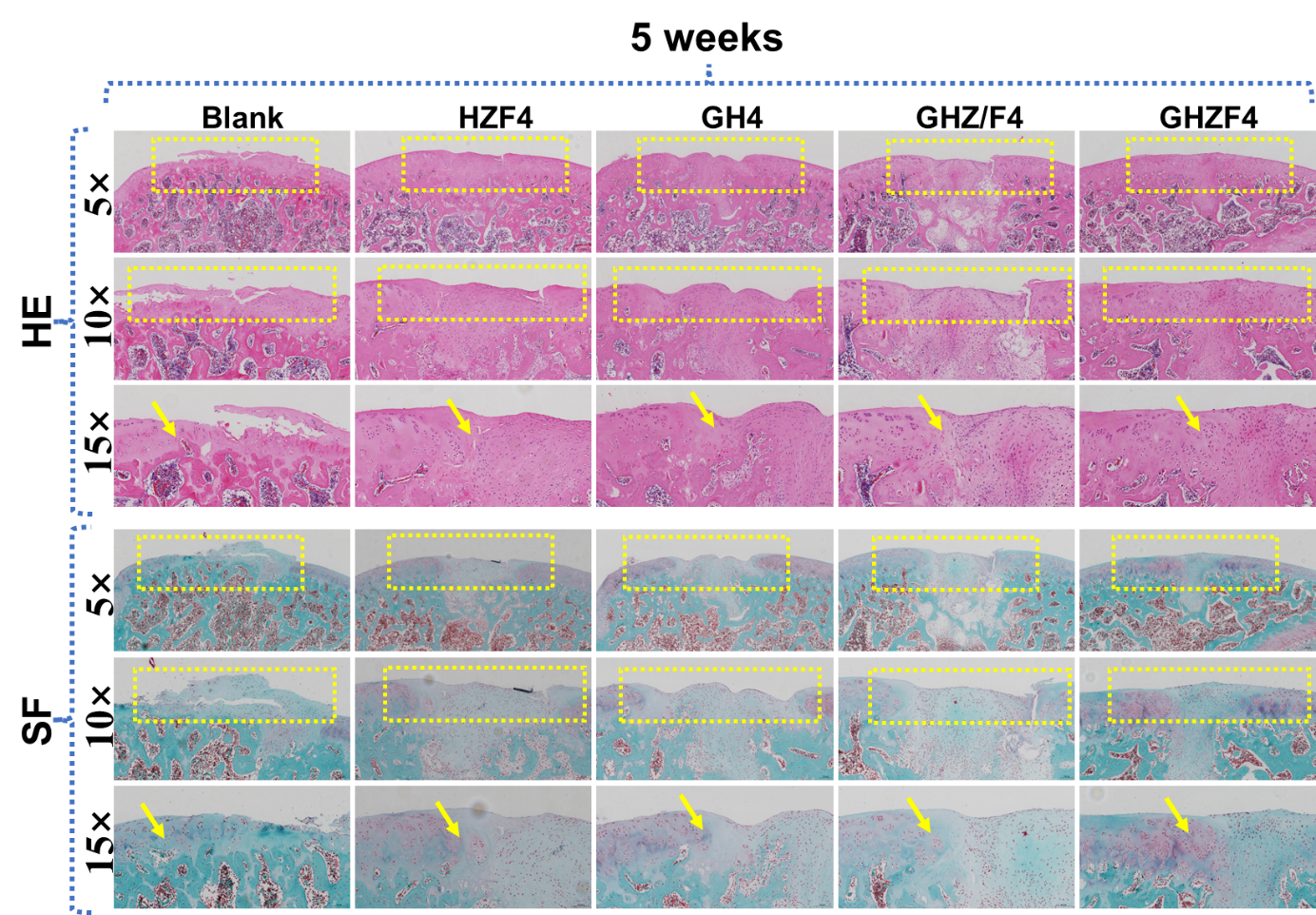


**Figure S16.** **H&E and Saf O/fast green staining of the regenerated osteochondral induced by hydrogels, shown at low and high magnification after 5 weeks.**


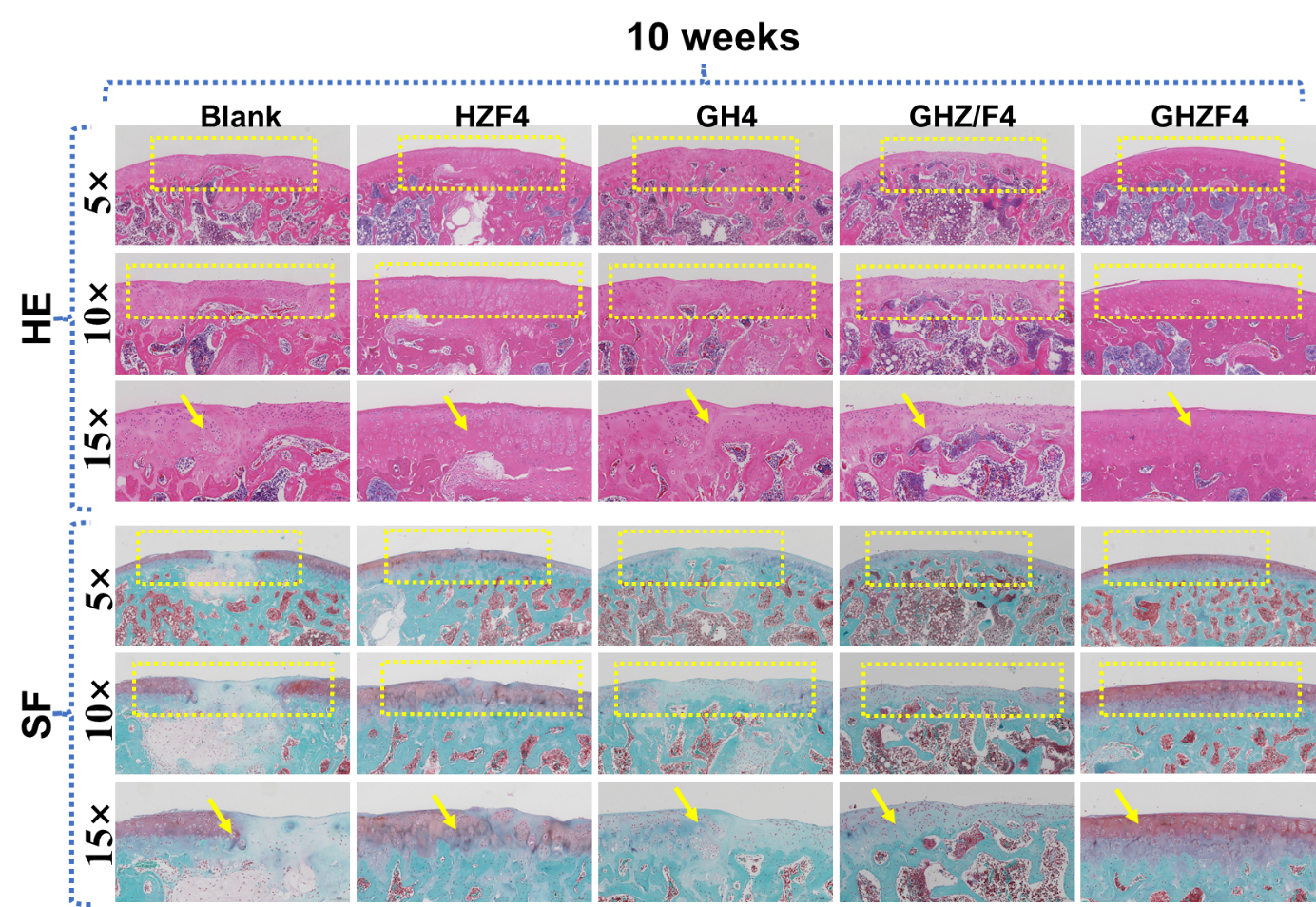


**Figure S17.** **H&E and Saf O/fast green staining of the regenerated osteochondral induced by hydrogels, shown at low and high magnification after 10 weeks.**


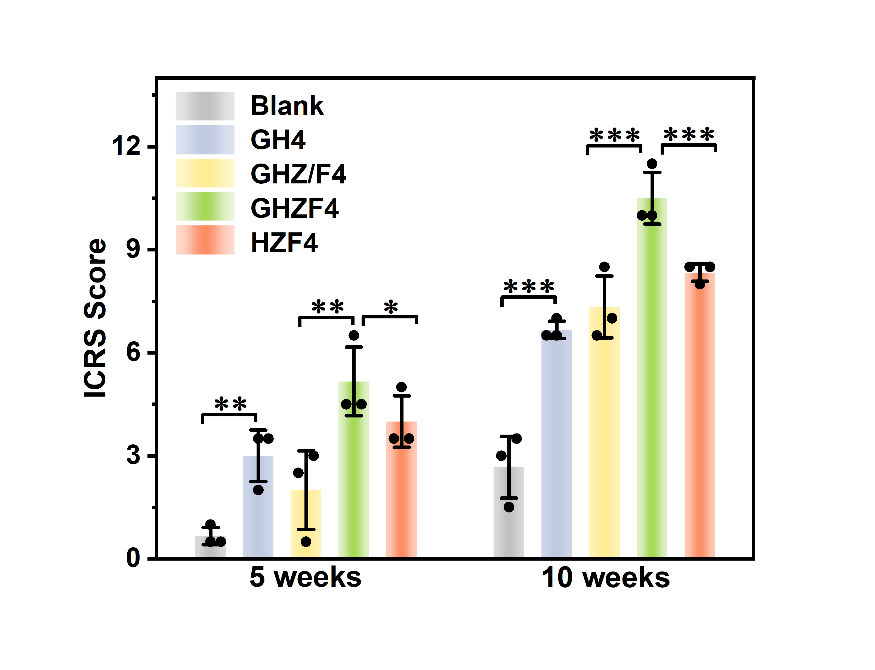


**Figure S18.** **The International Cartilage Repair Society Scoring System (ICRS) macroscopic scores for gross observations.**


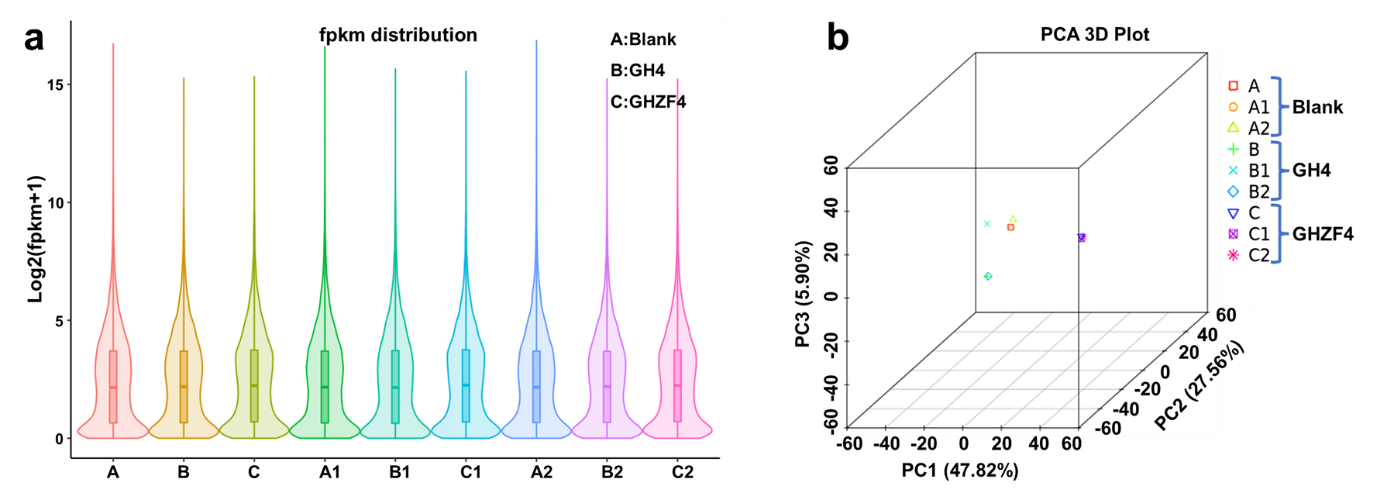


**Figure S19.****a)** Box plot depicting FPKM values; **b)** 3D image of principal component analysis of different samples.


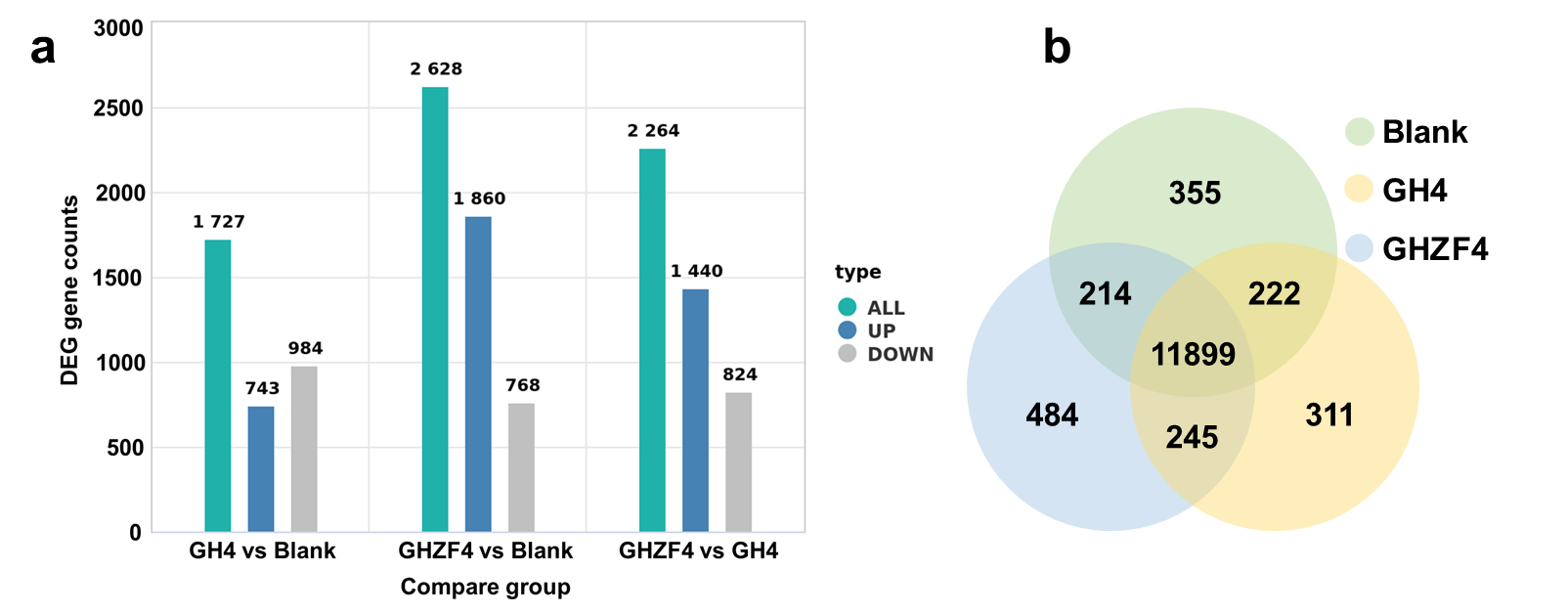


**Figure S20.** **a)** Histogram of the number of differentially expressed genes; **b)** Venn diagram of the number of differentially expressed genes in different groups.


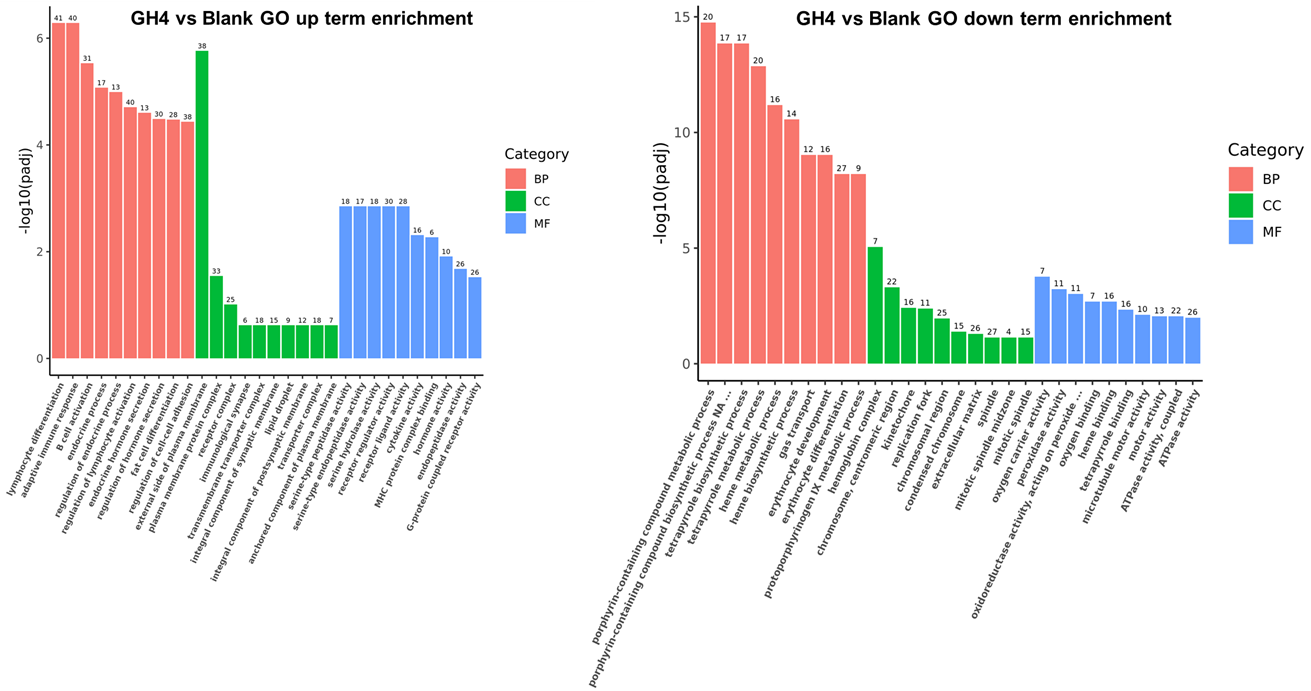


**Figure S21.****Enriched GO terms of GH4 versus Blank.**


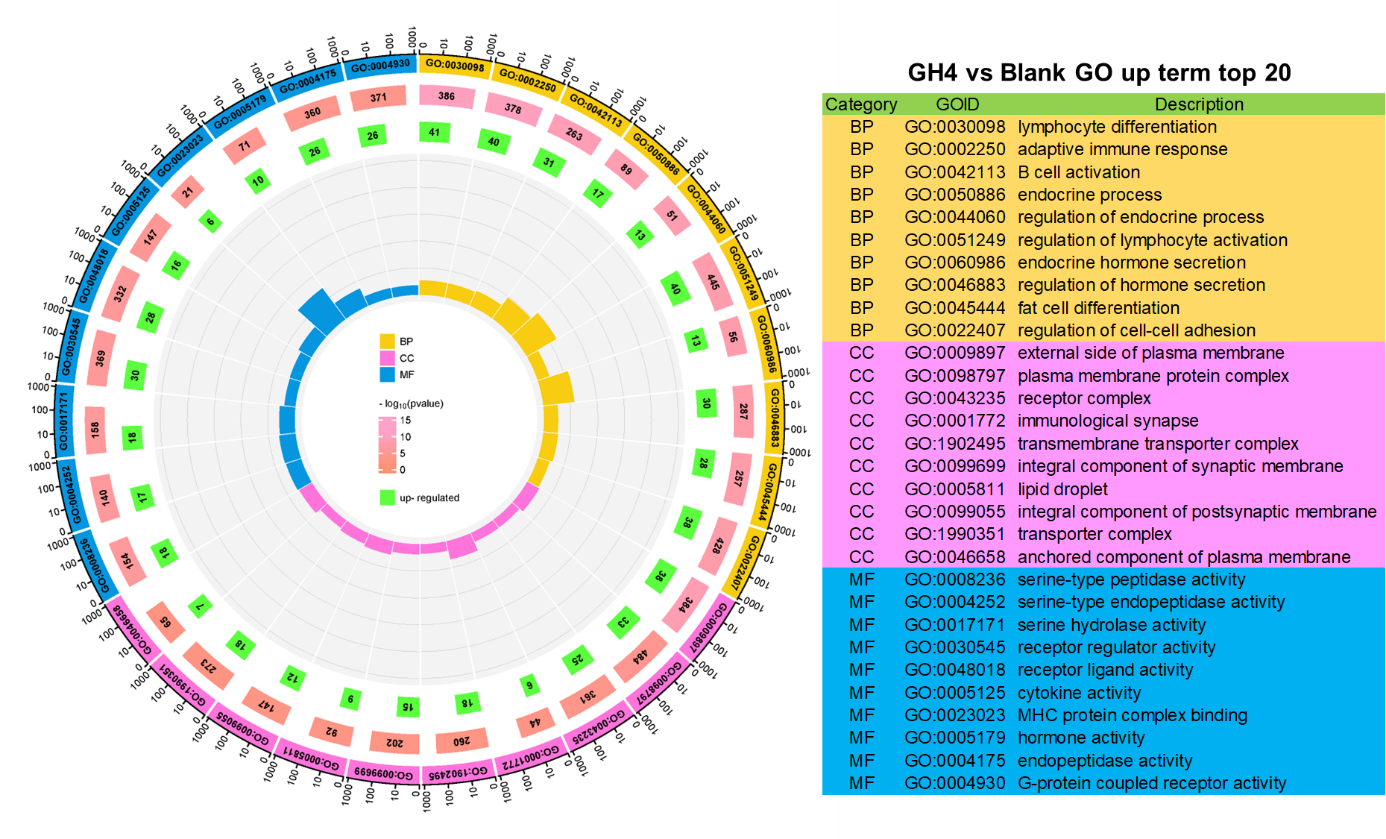


**Figure S22.** **Chordal diagram of GO-up enrichment of GH4 versus Blank.**


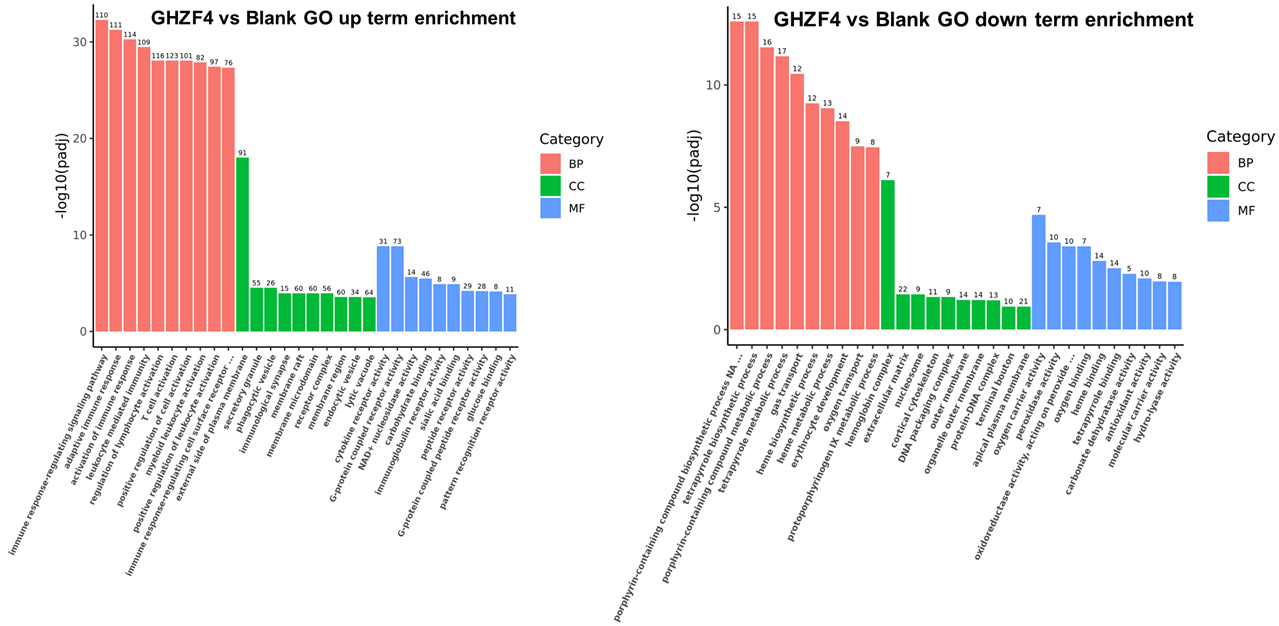


**Figure S23.****Enriched GO terms of GHZF4 versus Blank.**


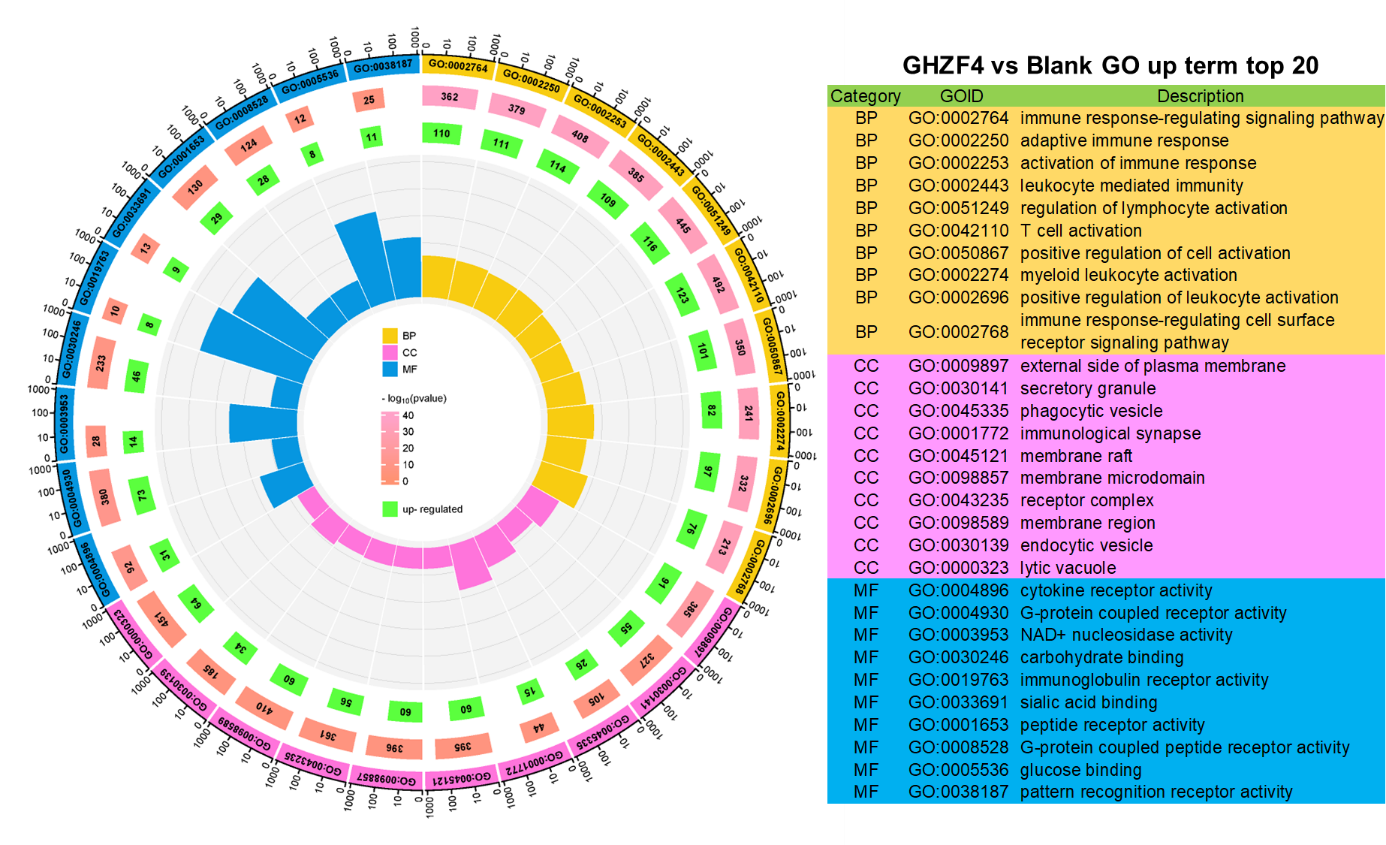


**Figure S24.** **Chordal diagram of GO-up enrichment of GHZF4 versus Blank.**


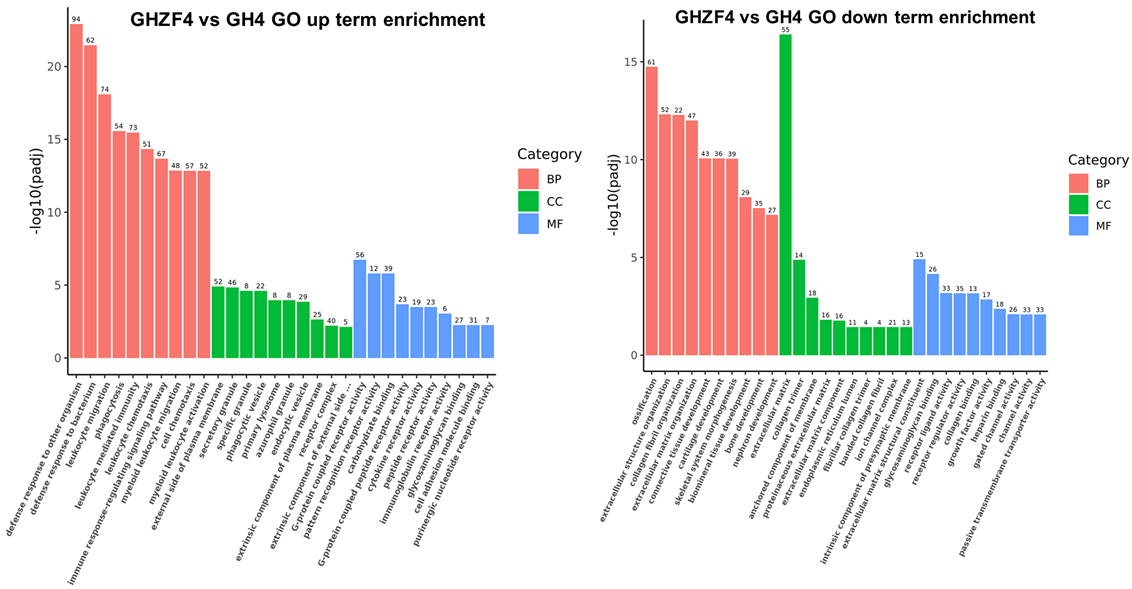


**Figure S25.****Enriched GO terms of GHZF4 versus GH4.**


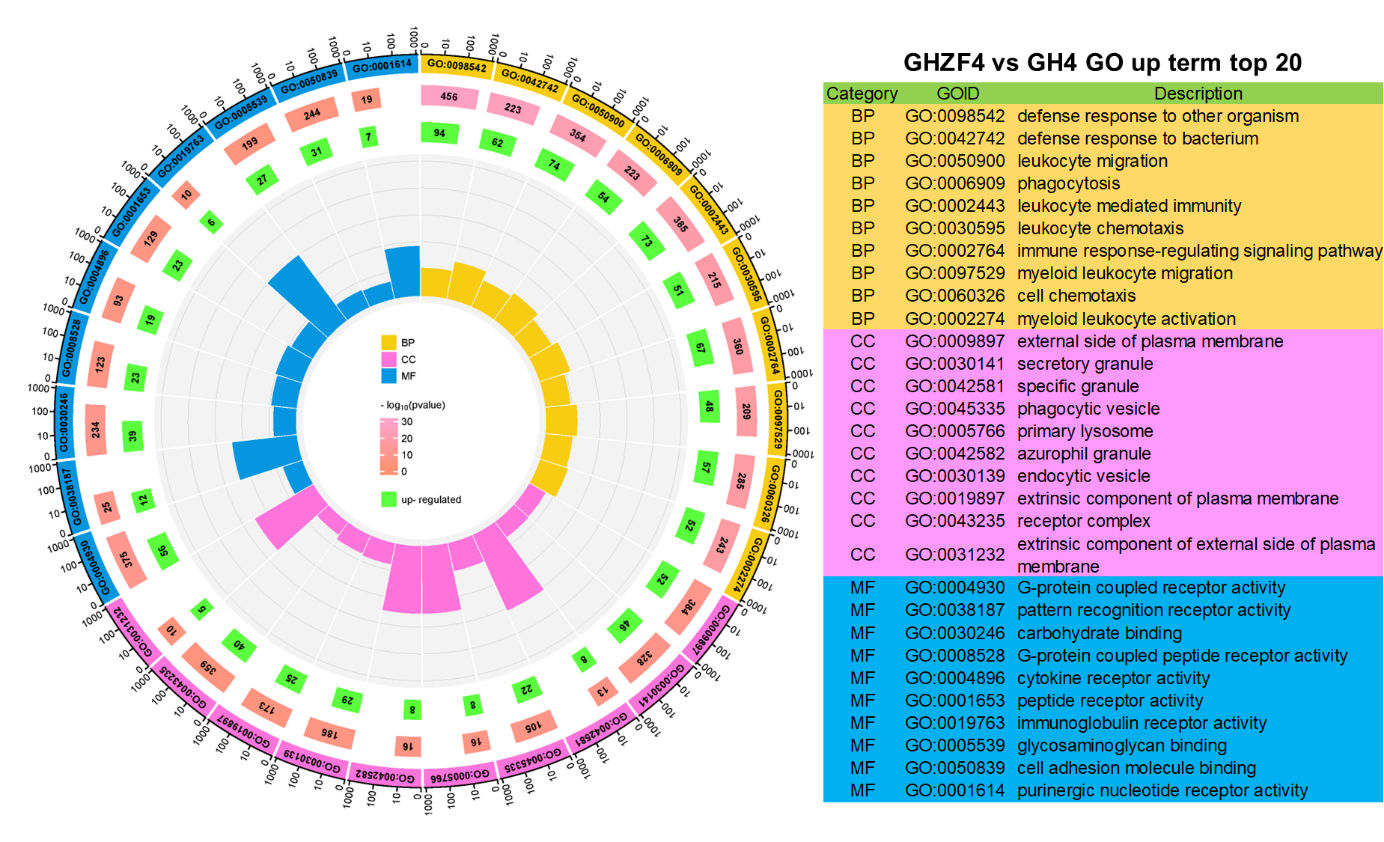


**Figure S26.****Chordal diagram of GO-up enrichment of GHZF4 versus GH4.**

**
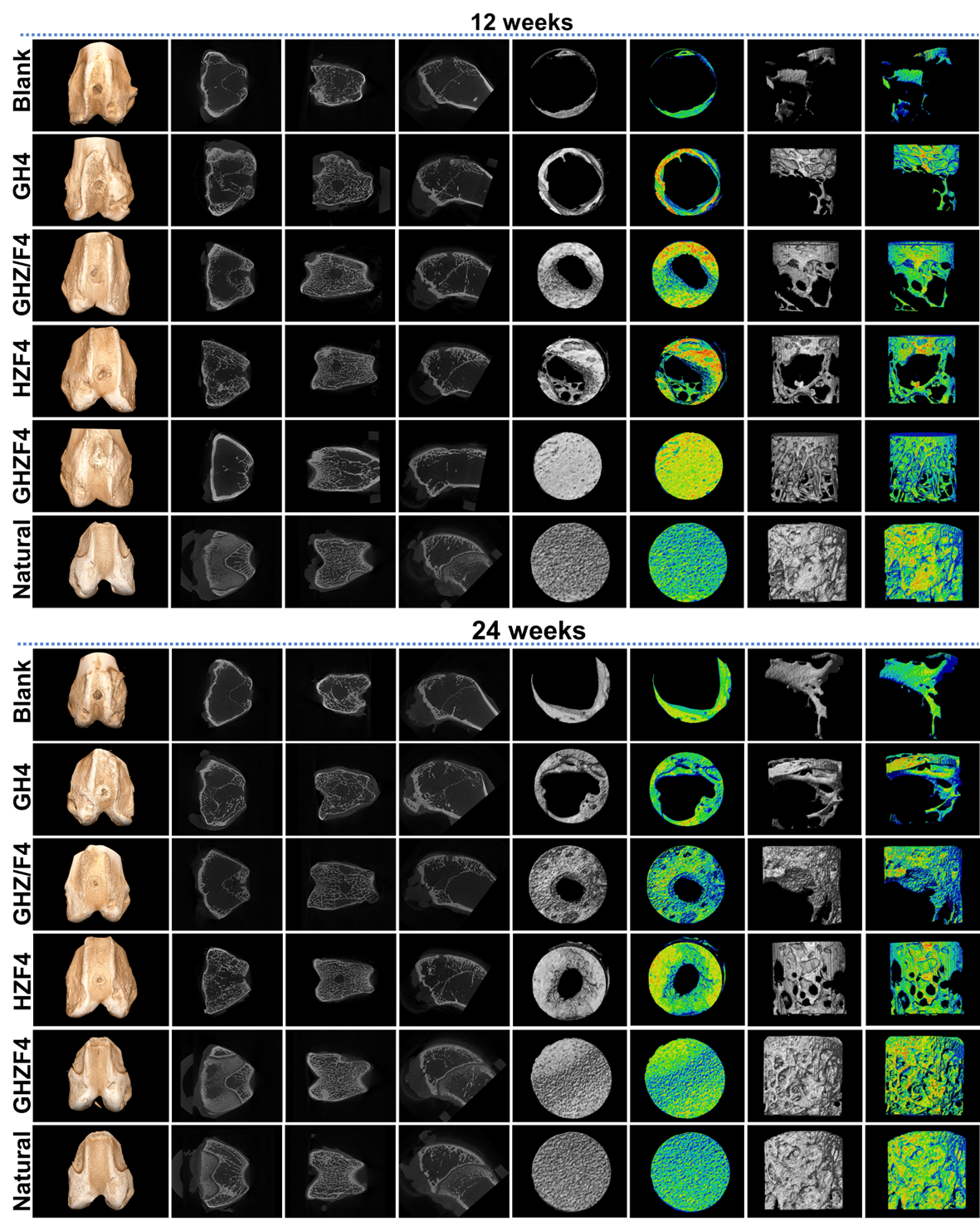
**

**Figure S27.****Reconstructed 3D Micro-CT images.**


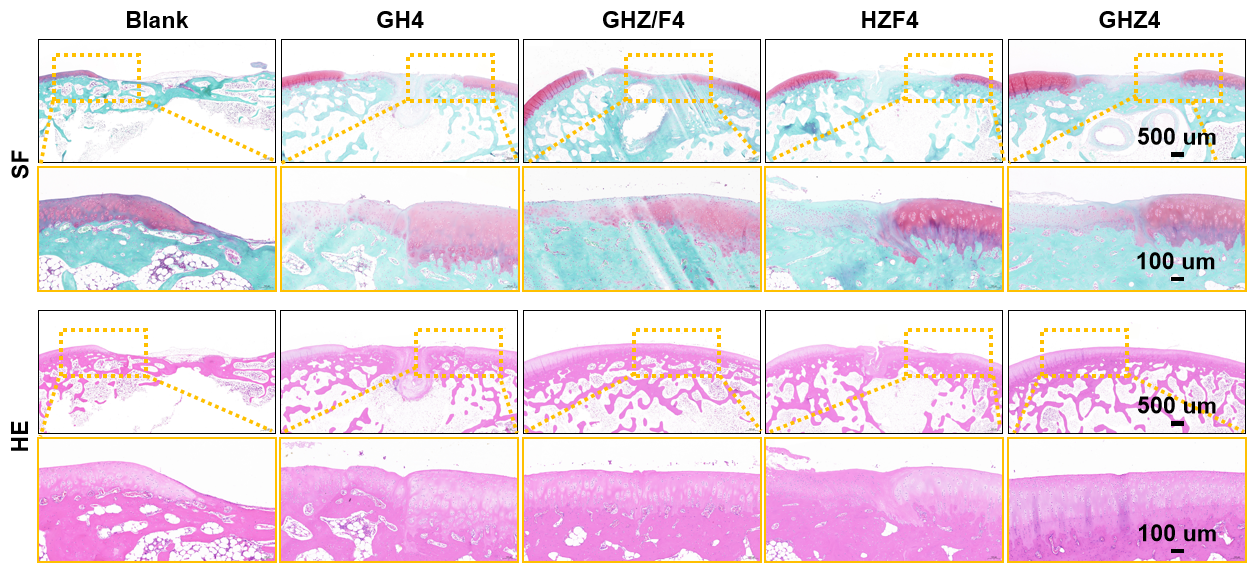


**Figure S28. Histological evaluation of rabbit osteochondral regeneration at 12 weeks post-implantation.** H&E and Safranin O/Fast Green staining of hydrogel-induced regenerated osteochondral tissues, presented at low and high magnification.

**Table S1.** **Primer sequences used for real-time qPCR**

| **Gene** | **Forward primers (5’-3’)** | | **Reverse primers (5’-3’)** |
| --- | --- | --- | --- |
| Actin | | CACCCAGCACAATGAAGATCAAGAT | CCAGTTTTTAAATCCTGAGTCAAGC |
| VEGF | | AGGGCAGAATCATCACGAAGT | GCACACAGGATGGCTTGAAGA |
| HIF-1α | | TGATTGCATCTCCATCTCCTACC | GACTCAAAGCGACAGATAACACG |
| β-actin | | TGCTATGTTGCCCTAGACTTCG | GTTGGCATAGAGGTCTTTACGG |
| RUNX2 | | TACCCAGGCGTATTTCAGATGAT | TGTAAGTGAAGGTGGCTGGATAGT |
| OPN | | GATGAACAGTATCCCGATGCCA | GTCTTCCCGTTGCTGTCCTGA |
| SOX9 | | CACAAGAAAGACCACCCCGA | TGCACGTCTGTTTTGGGAGT |
| ACAN | | AGGACTGCGTAGTGATGATCTGG | TAGCCTGTGCTTGTAGGTGTTGG |
| IL-1β | | GCATCCAGCTTCAAATCTCGC | TGTTCATCTCGGAGCCTGTAGTG |
| IL-6 | | TTCTTGGGACTGATGCTGGTG | CACAACTCTTTTCTCATTTCCACGA |
| IL-10 | | AATAAGCTCCAAGACCAAGGTGT | CATCATGTATGCTTCTATGCAGTTG |
| IL-1ra | | CTCTCCTTCTCATCCTTCTGTTTCA | TCCTTGTAAGTACCCAGCAATGAG |

**Table S2.** **Wakitani’s macroscopic scoring system**

| **Category** | **Item** | **Points** |
| --- | --- | --- |
| **Cell morphology** | Hyaline cartilage | 0 |
|  | Mostly hyaline cartilage | 1 |
|  | Mostly fibrocartilage | 2 |
|  | Mostly non-cartilage | 3 |
|  | Non-cartilage only | 4 |
| **Matrix staining** | Normal | 0 |
|  | Slightly reduced | 1 |
|  | Markedly reduced | 2 |
|  | No metachromatic stain | 3 |
| **Surface regularity (total smooth area compared with entire area of cartilage defect)** | Smooth (> 3/4) | 0 |
|  | Moderate (> 1/2 - 3/4) | 1 |
|  | Irregular (1/4 - 1/2) | 2 |
|  | Severely irregular (< 1/4) | 3 |
| **Thickness of cartilage** | > 2/3 | 0 |
|  | 1/3 - 2/3 | 1 |
|  | < 1/3 | 2 |
| **Integration of donor with host adjacent cartilage** | Both edges integrated | 0 |
|  | One end integrated | 1 |
|  | Neither edge integrated | 2 |
| **Neither edge integrated** |  | 14 |

Table S3. International Cartilage Repair Society (ICRS) macroscopic scoring system

| **Criteria** |  | **Points** |
| --- | --- | --- |
| **Degree of defect repair** | In level with surround cartilage | 4 |
|  | 75% repair of defect depth | 3 |
|  | 50% repair of defect depth | 2 |
|  | 25% repair of defect depth | 1 |
|  | 0% repair of defect depth | 0 |
| **Integration to border zone** | Complete integration with surrounding cartilage | 4 |
|  | Demarcating border < 1 mm | 3 |
|  | 3/4 of graft integrated, 1/4 with a notable border > 1 mm width | 2 |
|  | 1/2 of graft integrated with surrounding cartilage, 1/2 with a notable border > 1 mm | 1 |
|  | From no contact to 1/4 of graft integrated with surrounding cartilage | 0 |
| **Macroscopic appearance** | Intact smooth surface | 4 |
|  | Fibrillated surface | 3 |
|  | Small, scattered fissures or cracks | 2 |
|  | Several, small or few but large fissures | 1 |
|  | Total degeneration of grafted area | 0 |
| **Overall Repair Assessment** | | |
| **Grade I** | **Normal** | **12P** |
| **Grade II** | **Nearly normal** | **8-11P** |
| **Grade III** | **Abnormal** | **4-7P** |
| **Grade IV** | **Severely abnormal** | **1-3P** |
